# Gli1-mediated tumor cell-derived bFGF promotes tumor angiogenesis and pericyte coverage in non-small cell lung cancer

**DOI:** 10.1186/s13046-024-03003-0

**Published:** 2024-03-16

**Authors:** Xueping Lei, Zhan Li, Manting Huang, Lijuan Huang, Yong Huang, Sha Lv, Weisong Zhang, Zhuowen Chen, Yuanyu Ke, Songpei Li, Jingfei Chen, Xiangyu Yang, Qiudi Deng, Junshan Liu, Xiyong Yu

**Affiliations:** 1 Guangzhou Municipal and Guangdong Provincial Key Laboratory of Molecular Target & Clinical Pharmacology, the NMPA and State Key Laboratory of Respiratory Disease, School of Pharmaceutical Sciences &The Fifth Affiliated Hospital, Guangzhou Medical University, Guangzhou, 511436 People’s Republic of China; 2 Zhongshan Hospital of Traditional Chinese Medicine, Affiliated to Guangzhou University of Traditional Chinese Medicine, Zhongshan, 528400 PR China; 3 GMU-GIBH Joint School of Life Sciences, Joint Laboratory for Cell Fate Regulation and Diseases, The Guangdong-Hong Kong-Macau, Guangzhou Medical University, Guangzhou, 511436 PR China; 4 School of Traditional Chinese Medicine, Southern Medical University, Guangzhou, 510515 People’s Republic of China; 5 Guangdong Provincial Key Laboratory of Chinese Medicine Pharmaceutics, Guangzhou, 510515 People’s Republic of China

**Keywords:** Gli1, NSCLC, Angiogenesis, bFGF, Pericyte

## Abstract

**Background:**

Tumor angiogenesis inhibitors have been applied for non-small cell lung cancer (NSCLC) therapy. However, the drug resistance hinders their further development. Intercellular crosstalk between lung cancer cells and vascular cells was crucial for anti-angiogenenic resistance (AAD). However, the understanding of this crosstalk is still rudimentary. Our previous study showed that Glioma-associated oncogene 1 (Gli1) is a driver of NSCLC metastasis, but its role in lung cancer cell-vascular cell crosstalk remains unclear.

**Methods:**

Conditioned medium (CM) from Gli1-overexpressing or Gli1-knockdown NSCLC cells was used to educate endothelia cells and pericytes, and the effects of these media on angiogenesis and the maturation of new blood vessels were evaluated via wound healing assays, Transwell migration and invasion assays, tube formation assays and 3D coculture assays. The xenograft model was conducted to establish the effect of Gli1 on tumor angiogenesis and growth. Angiogenic antibody microarray analysis, ELISA, luciferase reporte, chromatin immunoprecipitation (ChIP), bFGF protein stability and ubiquitination assay were performed to explore how Gli1 regulate bFGF expression.

**Results:**

Gli1 overexpression in NSCLC cells enhanced the endothelial cell and pericyte motility required for angiogenesis required for angiogenesis. However, Gli1 knockout in NSCLC cells had opposite effect on this process. bFGF was critical for the enhancement effect on tumor angiogenesis. bFGF treatment reversed the Gli1 knockdown-mediated inhibition of angiogenesis. Mechanistically, Gli1 increased the bFGF protein level by promoting bFGF transcriptional activity and protein stability. Importantly, suppressing Gli1 with GANT-61 obviously inhibited angiogenesis.

**Conclusion:**

The Gli1-bFGF axis is crucial for the crosstalk between lung cancer cells and vascular cells. Targeting Gli1 is a potential therapeutic approach for NSCLC angiogenesis.

**Supplementary Information:**

The online version contains supplementary material available at 10.1186/s13046-024-03003-0.

## Introduction

Non-small cell lung cancer (NSCLC), the most common type of lung cancer, has a high mortality rate [1]. Although many improvements have been made in NSCLC therapy, including the introduction of targeting epidermal growth factor receptor (EGFR) mutations or ALK rearrangements, immunotherapy and surgery, the prognosis of NSCLC patients is still dismal. These therapeutic approaches have demonstrated unprecedented survival benefits in selected patients. However, the emergence of drug resistance has blocked their further application. Currently, the survival rate of patients with NSCLC is only approximately 20%. Hence, it is necessary to identify prognostic biomarkers and develop novel therapeutic approaches for NSCLC [2, 3].

NSCLC tumors are a highly vascularized tumor. A high microvessel density often indicates a poor clinical prognosis in NSCLC patient [4, 5]. Tumor angiogenesis is necessary for the occurrence, development and metastasis of NSCLC. Thus, antiangiogenic approaches have been developed for NSCLC therapy. Many antiangiogenic agents targeting the VEGF-A/VEGFR2 signaling pathway, such as bevacizumab, ramucirumab and nintedanib, are currently used for the treatment of NSCLC patients or are in development [6–8]. Combinations of these angiogenesis inhibitors with checkpoint inhibitors, chemotherapy and EGFR inhibitors have significantly improved the survival of NSCLC patients [9, 10]. Whereas, only a subset of patients benefits from these angiogenesis inhibitors, and their effects are generally incomplete and temporary. In addition, many patients exhibit antiangiogenic drug (AAD) resistance. Therefore, further elucidation of the underlying mechanisms responsible is necessary for the development of effective therapeutic approaches for NSCLC [6, 7].

The majority of AADs used in the clinic primarily affect tumor vascular endothelial cells, with no effects on tumor cells and other perivascular cells. Many patients exhibit patients suffer from resistance after AAD therapy. Recently, crosstalk between tumor cells and vascular cells was identified as a crucial factor for AAD resistance and angiogenesis [11]. During AAD therapy, cancer cells accelerate the acquisition of AAD resistance by activating fatty acid oxidation metabolic [12]. Glioblastoma cells promote bevacizumab resistance by upregulating YKL-40 and ZEB1 expression, and mesenchymal transition [13]. These findings suggest that tumor cell-perivascular cell interaction was crucial for AAD resistance. However, our understanding of intercellular crosstalk between lung cancer cells and perivascular cells is still rudimentary [14, 15].

Glioma-associated oncogene 1 (Gli1) is a member of the zinc finger protein family. It is crucial for the activation of the canonical hedgehog (Hh)/Gli1 signaling pathway, which is frequently activated in many cancers, including lung cancer, breast cancer and basal-cell carcinoma. When it is activated, Gli1 translocates from the cytoplasm to the nucleus, leading to the expression of various target genes and signal transduction through downstream signaling pathways [16]. Gli1 is a driver of cell survival and CSC stemness features [17]. Our previous study showed Gli1 was upregulated in NSCLC and was a critical driver of NSCLC metastasis [18]. Moreover, inhibition of Gli1 nuclear translocation obviously suppressed angiogenesis in NSCLC [19], indicating that Gli1 may be involved in angiogenesis of NSCLC. However, whether Gli1 is involved in the intercellular crosstalk between lung cancer cells and vascular cells is still unclear.

Herein, we confirmed that Gli1 promoted tumor angiogenesis by regulating the crosstalk between lung cancer cells and vascular cells. Gli1 overexpression in NSCLC cells enhanced the migration, invasion, tube formation of endothelial cells and increased the microvessel density in mouse xenografts. Whereas genetic inhibition of Gli1 in NSCLC cells resulted in the opposite effects on endothelial cells. Gli1 modulation also had a similar effect on pericyte coverage. Angiogenic antibody microarray analysis identified bFGF as a downstream regulator of Gli1. Additionally, bFGF was found to be critical for the Gli1-mediated enhancement of angiogenesis. Moreover, mechanistic research revealed that Gli1 increased bFGF protein expression through promoting bFGF transcriptional activity and prolonging bFGF protein stability. Finally, we also showed that therapeutic blockade of Gli1 using GANT-61 obviously suppressed tumor angiogenesis. The findings of this study suggests that Gli1 is a promising therapeutic target for attenuating angiogenesis in NSCLC. This study also provides intriguing insights into the crosstalk between NSCLC cells and vascular cells during the regulation of tumor angiogenesis.

## Materials and methods

### Regents

Endothelial cell medium (ECM) and pericyte medium (PM) were products of ScienCell Research Laboratories (San Diego, CA, USA). Antibodies against Ki67 (#12,202), α-SMA (#19,245), bFGF (#20,102), FGFR1 (#76,123), FGFR2 (#23,328), β-actin (#23,328) and HRP-conjugated anti-rabbit IgG (#7074) were obtained from Cell Signaling Technology (Danvers, MA, USA). Antibodies against Gli1 (AF3455) and CD31 (AF3628), and a Proteome Profiler Human Angiogenesis Array Kit (ARY007) were obtained from R&D Systems (Minneapolis, MN, USA). Alexa Fluor 594-conjugated donkey anti-rabbit IgG (ab150064) and Alexa Fluor488- conjugated donkey anti-mouse IgG (ab150073) were obtained from Abcam (Cambridge, UK). GANT-61, cycloheximide (CHX) and MG132 were purchased from TargetMol (Shanhai, China). Matrigel was obtained from BD Biosciences (Franklin Lakes, NJ). Recombinant human bFGF was purchased from BioVision (Palo Alto, CA, USA). A ChIP PCR kit was obtained from Merck Millipore (Billerica, MA, USA). A dual luciferase reporter gene assay kit was provided by Promega (Madison, WI, USA). Rhodamine-labeled lysinated dextran (70-kDa) was product from Thermo Fisher Scientific (Waltham, MA, USA). PKH 26, PKH 67 and other reagents were obtained from Sigma-Aldrich (St. Louis, MO, USA).

### Patient specimens and immunohistochemistry (IHC)

Primary NSCLC tissues and adjacent noncarcinoma samples were from patients who underwent curative resection at the Sixth Affiliated hospital of Guangzhou Medical University. These patients were histopathologically diagnosed with NSCLC and had available survival. All patients have signed written informed consent prior to tissue collection. A cohort composed of 90 pairs of NSCLC tissues and the corresponding noncarcinoma tissues was constructed by Shanghai Xinchao Biotech (Shanghai, China). A tissue microarray was applied for the IHC assay to detect bFGF expression. Tumor cells stained with Gli1 or bFGF were calculated per field of view. The expression of bFGF was calculated based on staining intensity and distribution [18]. The correlation between Gil1 and expression was analyzed based on the IHC score.

### Animals

Five-week-old male BALB/c (nu/nu) mice and adult female Sprague–Dawley (SD) rats were obtained from Hua Fukang Experimental Animal Center (Beijing, China). All experimental procedures conducted in animal studies were approved by the laboratory animal ethics committee of Guangzhou Medical University.

### Cell lines and culture conditions

Human umbilical vein endothelial cells (HUVECs), human microvascular endothelial cell line-1 (HMEC-1) cells and human brain vascular pericytes (HBVPs) were provided by ScienCell Research Laboratories (Carlsbad, CA). The human NSCLC lines NCI-H1299, NCI-H1703, NCI-H460 and A549 cells were provided by American Type Culture Collection (ATCC, Manassas, Virginia). HUVECs were cultured with ECM. HBVPs were maintained in PM. HMEC-1 cells were cultured with DMEM supplemented with 10% FBS. The human NSCLC cell lines were cultured in RPMI 1640 medium containing 10% FBS and 1% penicillin–streptomycin (v/v). All the cells were maintained in a humidified incubator containing 5% CO_2_.

### Conditioned medium (CM) generation

The stable cell lines including NCI-H1299^shNC^/NCI-H1299^shGli1^, NCI-H1703^shNC^/NCI-H1703^shGli1^, A549^NC vector^/A549^Gli1vector^, and NCI-H460^NC vector^/NCI-H460^Gli1vector^ cells were established in our previous study [18]. These cells were maintained in RPMI 1640 medium containing 10% FBS and 1% penicillin–streptomycin (v/v). When the cell density reached approximately 80%, the medium was replaced with fresh medium containing 50% RPMI 1640 medium and 50% ECM (v/v) for 24 h to generate the CM of endothelial cells. And the fresh medium containing 50% RPMI 1640 medium and 50% PM (v/v) for 24 h to generate the CM of HBVPs. The media were collected and filtered through 0.22 μm filter (SLCPR33RB, Millipore). Then the supernatants were aliquoted and frozen at -80 °C. The CM was used for further endothelia cells or pericytes related experiments.

### Angiogenic antibody microarray analysis

The human angiogenic antibody microarray was obtained from R&D. The CM of A549^NC vector^/A549^Gli1 vector^ cells was collected and used for angiogenesis factors detection following the manufacturer’s instructions. Briefly, angiogenic antibody membranes were incubated with blocking buffer, and were then incubated with 1.5 mL of CM from A549^NC vector^/A549^Gli1 vector^ cells at 4 °C overnight. After washing with PBS, the antibody membranes were incubated with streptavidin–horseradish peroxidase solution for 1.5 h at room temperature. Protein expression was detected by an enhanced chemiluminescence (ECL) kit and a ProScan HT Microarray Scanner (PerkinElmer, MA). The densities of individual spots were analyzed using the Scan Array Express software.

### ELISA assay

ELISA assay was performed to detect the bFGF concentration in the CM of four paired cell lines with Gli1 overexpression or knockdown. These cells were cultured with the corresponding culture medium for 48 h. After that, the medium was collected and centrifuged. The supernatant was used for ELISA assay according to the manufacturer’s instructions.

### Wound healing assay

The cells were plated into 6-well plates and incubated until they were approximately 90% confluent. The cells layer was then scratched using a 10 μL sterile pipette tip to generate a wound and then exposed to CM from NSCLC cells for 8 h. The migration ability was indicated by the width of the gap between two edges of the wound, which was measured using a microscope at 0 h and 12 h.

### Transwell migration and invasion assays

HUVECs and HMEC-1 cells (2 × 10^4^) were incubated with NSCLC cell CM for 48 h. Then, the cells were collected, suspended in 100 µL of serum-free medium and seeded in the upper chamber of a Transwell insert. The lower chamber was filled with 650 µL of fresh medium and the cells were incubated for 24 h. Then the cells were fixed with 4% paraformaldehyde and stained with crystal violet solution. After the cells on the upper surface of the chamber membrane were removed, the cells attached to the lower surface of the chamber membrane were observed using a light microscope.

For the Transwell invasion assay, the procedures were largely similar to those used for the Transwell migration assay, except that the chambers membranes were precoated with Matrigel.

### Tube formation assay

The wells of 96-well microwell plates were coated with 50 µL-Matrigel and the plates were placed at 37 °C for solidification. Endothelial cells that were exposed to the indicated CM were seeded in the 96-well plates at a density of 2 × 10^4^ cells per well. After incubation for another 6 h, the capillary-like structures were observed with a light microscope.

### Aortic ring assay

The aortic ring assay was performed in a 96-well plate with the wells precoated with 50 µL Matrigel. The thoracic aorta was isolated from female SD rats weighing 200 g in a sterile manner and then cut into approximately 1 mm long rings. Each ring was seeded in Matrigel-coated 96-well plates, and covered with another 80 µL of Matrigel. After 2 h, 100 μL of CM were added to the rings. The CM was replaced every two days. The rings were observed and photographed on Day 8 with microscope (Olympus IX71, Japan). The number of microvessels was quantitated with the image analysis program IPP 6.0.

### HBVP adhesion assay

The HBVPs were exposed to CM for 48 h, and were collected and placed at a 24-well plate with a density of 1 × 10^5^ cells/well. After culturing for 1 h, the cells were sequentially fixed with 4% paraformaldehyde and stained with 0.1% crystal violet in sequence. The adherent cells were observed and captured using an Olympus BX53 upright microscope. Image-Pro Plus 6.0 was used to analyze the adhesion ability of HBVPs.

### Coculture of tubule-like structures of endothelial cells and pericytes

HUVECs labeled with PKH26 (red) were placed in Matrigel-coated 96-well plates at a density of 3 × 10^4^ cells per well, and incubated for 2 h to allow the cells to form a capillary network. HBVPs pretreated with CM from NSCLC cells were labeled with PKH67 (green) and seeded into the endothelial capillary network at a density of 1 × 10^4^ cells per well. The tube structures and recruitment of HBVPs were observed and photographed using an EVOS XL Core imaging system at 0 h, 1 h and 10 h. The time of HBVP addition was set as 0 h.

### In vivo* mouse xenograft assay*

The four pairs of stable NSCLC cells were subcutaneously injected into the left flanks of 5–6-week-old male nude (BALB/cA-nu) mice (1 × 10^7^ cells per mice). When the tumor volume reached approximately 50 mm^3^, the tumors were measured using a caliper and the tumor volume calculated with the formula (0.5 × width^2^ × length) for totally 22 days. The mice were then anesthetized and the tumors were excised, weighed and photographed. Subsequently, the tumors were fixed using 4% paraformaldehyde for further histological and immunohistochemical assays.

To assess the antiangiogenic effect of GANT-61, NCI-H1299 cells were subcutaneously inoculated into the left flanks of mice. The tumor bearing mice were randomly divided into the vehicle group and 50 mg/kg GANT-61group with 6 mice per group once the tumor volume reached approximately 50 mm^3^. The mice were intraperitoneally injected with saline or 50 mg/kg GANT-61 every other day for a total of 24 days.

### Immunofluorescence assay of OCT-embedded tissues

Tumor tissues fixed in paraformaldehyde were frozen in OCT (Tissue Tek, USA) and subsequently cut into 5-µm-thick sections. The tumor sections were permeabilized with 0.25% Triton-X-100, and then incubated with anti-CD31 and anti-α-SMA primary antibodies. After washing with PBS, the tumor sections were labeled with the corresponding Alexa Fluor 488- or Alexa Fluor 594-conjugated secondary antibodies. DAPI staining was applied for nuclear labeling. Images were acquired with a confocal microscope (LSM 800, Zeiss).

### Tumor vascular permeability assay

Tumor vascular permeability was evaluated as previously described [20]. In brief, the mice were intravenously injected with rhodamine-labeled dextran (70-kDa). Twenty-four hours later, the mice were anesthetized. The tumors were excised and fixed, and following by frozen in OCT. Dextran in tumor Sect. (5 µm) was observed and photographed using a confocal microscope. The data are presented as the mean fluorescence intensity values calculated by ImageJ software (National Institutes of Health).

### Luciferase reporter assay

The sequence of the bFGF promoter (1179 bp) was and inserted into the pGL3-Basic-Luc vector to generate the pGL3-bFGF-promoter^WT−Luc^ vector. The two mutated bFGF promoter fragments (from bp -180 to -168 and from bp + 70 to + 82) were constructed by RT‒qPCR and were then cloned and inserted into the same reporter vector. The pGL3-bFGF-promoter^Mut1−Luc^ plasmid (“TTTGGGTGGTGC” mutated to “CCCAAACAACAT”) and the pGL3-bFGF-promoter^Mut2−Luc^ plasmid (“TGTGGGGGGTGG” mutated to “CACAAAAAACAA”) were constructed using a site-directed mutagenesis kit (Genechem, China) according to the manufacturer's instructions. The 293 T cells were seeded in 24-well plates and transfected as indicated with the pGL3-bFGF-promoter^WT−Luc^/ pGL3-bFGF-promoter^Mut1−Luc^, pGL3-bFGF-promoter^Mut2−Luc^/pGL3-bFGF-promoter^Mut1+Mu2−Luc^ and Renilla pRL-TK plasmids using Lipofectamine 3000 following with the manufacturer’s instructions. Forty-eight hours later, the cells were collected and lysed using cold lysis buffer (Promega). The cell lysates were collected and added to a 96-well plate illuminometer to measure the fluorescence intensity by a microplate reader. Firefly luciferase activity was normalized to Renilla luciferase activity as the internal control.

### Chromatin immunoprecipitation (ChIP) assay

A ChIP assay kit was employed to fulfill ChIP assay following with manufacturer’s protocol. In short, the cells were collected and exposed to 1% formaldehyde to crosslink chromatin-associated proteins to DNA. Subsequently, the nuclear pellet was resuspended in ChIP Buffer and then applied for sonication. After incubation of the nuclear pellet suspension with anti-Gli1 antibody- and anti-IgG-conjugated Dynabeads protein A/G at 4 °C overnight, DNA‒protein complexes were purified using DNA purification magnetic beads and then eluted with DNA elution buffer. The expression levels of the target genes were determined by RT‒qPCR assay. The primers were displayed in Supporting Table 1.

### bFGF protein stability assay

The stability of the bFGF protein was measured by CHX pulse-chase experiments. Briefly, cells were exposed to 2 μM CHX for 0 h, 0.5 h, 1 h, 2 h, 4 h and 6 h. Then, the cells were harvested, lysed with RIPA buffer and then applied for Western blottingto measure the bFGF protein level.

### Ubiquitination assay

The cells were treated with the proteasome inhibitor MG132 (0.5 μM) for 6 h and lysed with immunoprecipitation lysis buffer. Proteins (500 μg per sample) were incubated with anti-bFGF antibody- or normal IgG-bound Protein A + G beads at 4 °C overnight. The reaction was terminated by the addition of loading buffer. Ubiquitination was analyzed by Western blotting with antibodies against bFGF and ubiquitin.

### Statistical analysis

Statistical analyses were performed with GraphPad Prism 7 (GraphPad, San Diego, CA, USA) and the data were displayed as the means ± SDs. Mos of in vitro experiments were conducted three times independently. Comparisons between two groups were analyzed using an unpaired t test, and one-way analysis of variance (ANOVA) was used to evaluate the statistical significance of differences among three or more groups. *p* < 0.05 was considered to indicate statistical significance.

## Results

### Gli1 overexpression in NSCLC cells promoted tumor angiogenesis

**Fig. 1:**
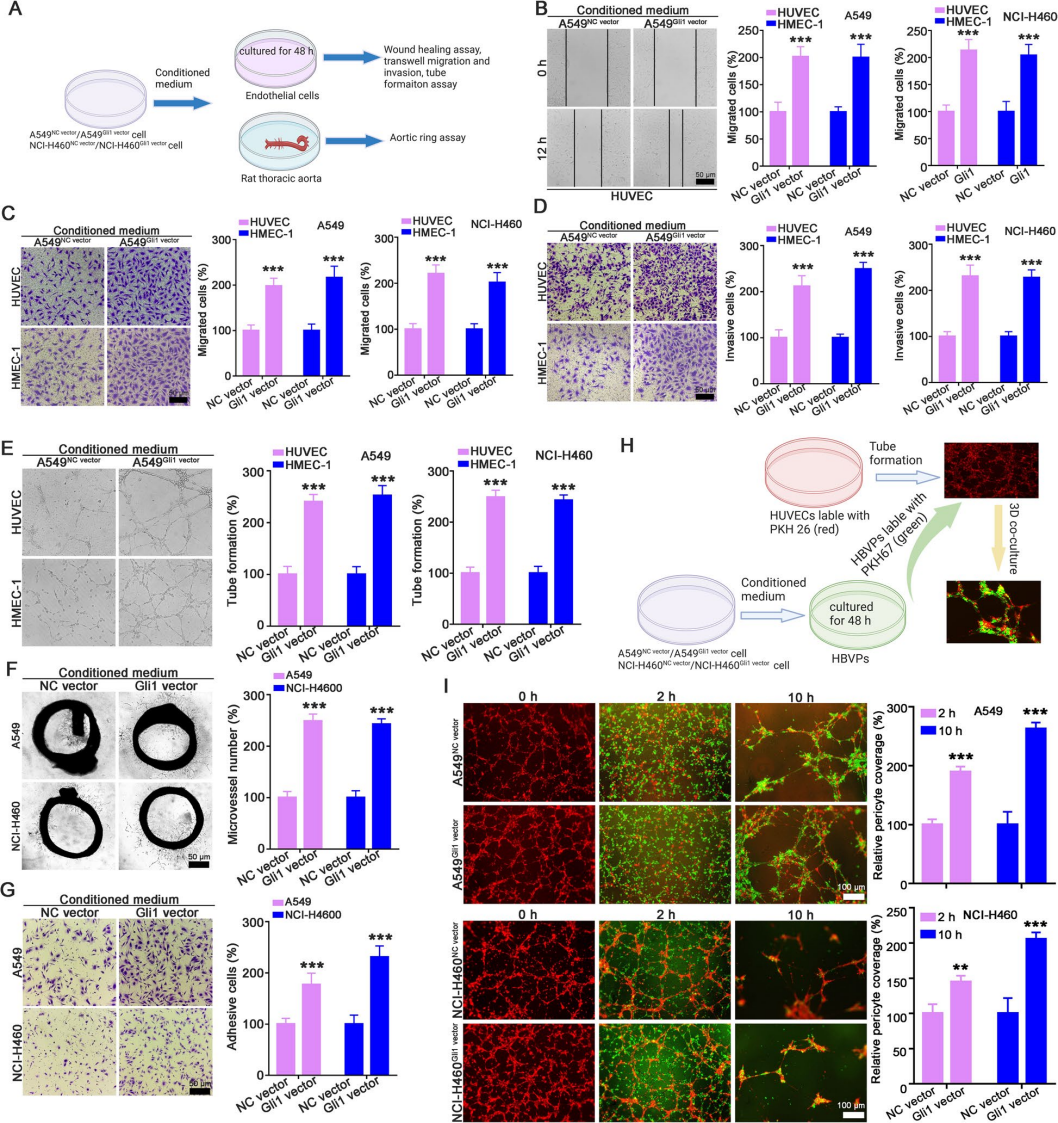
The CM of Gli1-overexpressing NSCLC cells promoted angiogenesis in vitro and ex vivo. **A** Flowchart of evaluation the effect of Gli1-overexpressing A549 and NCI-H460 cells on endothelial cell and aorta rings. **B-E** The effect of CM from A549^Gli1 vector^ and NCI-H460^Gli1 vector^ cells on the migration, invasion and tube formation abilities of HUVECs and HMEC-1 cells. HUVECs and HMEC-1 cells were treated with CM for 48 h and subjected for wound healing (**B**), Transwell migration (**C**) and Tiranswell invasion (**D**), and tube formation assays (**E**). **F** Gli1-overexpressing NSCLC increased blood sprouting in aortic ring assay. **G** The CM of Gli1-overexpressing cells promoted the HBVP adhesion ability. **H** Schematic strategy of coculture tubule-like structure of endothelial cells and pericytes. **I** CM from Gli1-overexpressing cells enhanced the recruitment of HBVP to tubes formed by endothelial cell. All the data were showed as mean ± SD, *n* = 3. ^**^p < 0.01; ^***^p < 0.001 vs A549^NC vector^ or NCI-H460^NC vector^ cells

To clarify the function of Gli1 in angiogenesis, CM from A549^NC vector^/A549^Gli1 vector^ and NCI-H460^NC vector^/NCI-H460^Gli1 vector^ cells was used as a chemoattractant for naive HUVECs and HMEC-1 cells (Fig. 1A and Supporting Fig. 1). The wound-healing assay showed that compared with CM from A549^NC vector^ or NCI-H460^NC vector^ cells, CM from A549^Gli1 vector^ or NCI-H460^Gli1 vector^ cells significantly promoted the migration of endothelial cells (Fig. 1B and Supporting Fig. 2A). Concordantly, the Transwell migration and invasion assays demonstrated that endothelial cells exposed to CM from A549^Gli1 vector^ or NCI-H460^Gli1 vector^ cells exhibited enhanced migration and invasion abilities (Fig. 1C-D). And elevated lumen-forming ability of endothelial cells exposed to CM from A549^Gli1 vector^ or NCI-H460^Gli1 vector^ cells were also observed (Fig. 1E). Furthermore, the aortic ring assay demonstrated that the CM of A549^Gli1 vector^ and NCI-H460^Gli1 vector^ cells markedly augmented aortic ring sprouting (Fig. 1F). In addition, considering that pericyte coverage is critical for blood vessel maturation [21], we also evaluated the role of Gli1 in pericyte adhesion and recruitment. The pericyte adhesion assay showed that Gli1 overexpression in A549 cells and NCI-H460 cells significantly increased the adhesion ability of HBVPs (Fig. 1G). Consistently, the 3D coculture assay also showed that most of the HBVPs (green) educated by A549^Gli1 vector^ or NCI-H460^Gli1 vector^ cells had migrated to tube structures (red) at 2 h. The tubes had developed into stronger and more stable structures at 12 h (Fig. 1H-I).

**Fig. 2:**
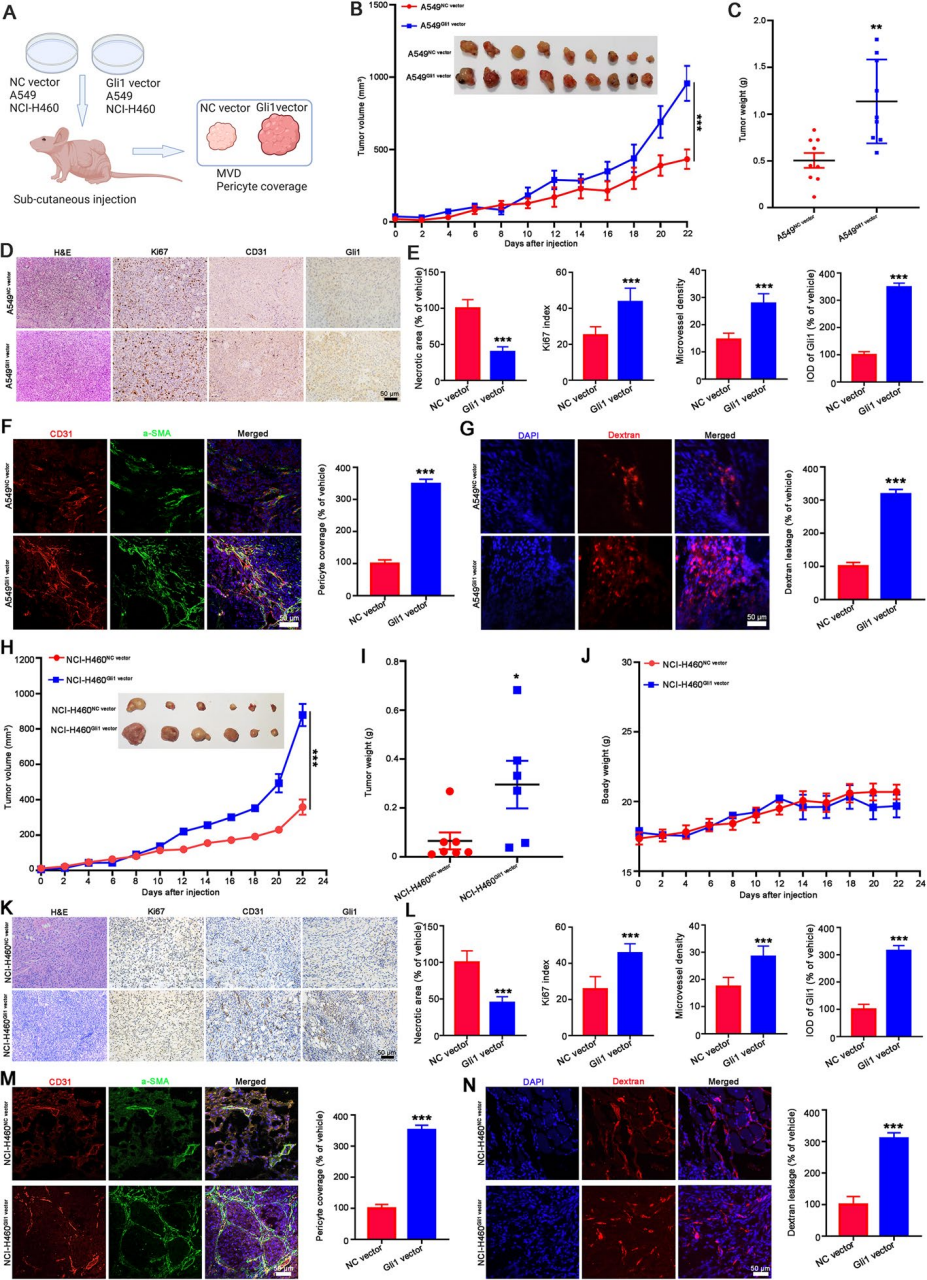
Gli1 overexpression promoted tumor angiogenesis and increased pericyte coverage of blood vessel. **A** Flowchart of investigating the effect of Gli1 on tumor angiogenesis and pericyte coverage. **B**, **C** Gli1 overexpression in A549 cells promotes tumor growth. The A549^NC vector^ and A549^Gli1 vector^ cells were subcutaneously injected to establish a xenograft model. The tumor volumes were measured every two days. At the end of the experiment, the tumors were removed, weighted, photographed and then subjected for further pathological analysis. The tumor volume curve and tumor image were presented in (**B**), the tumor weight statistics was showed in (**C**). **D**, **E** The results of H&E staining and IHC staining of Ki67, CD 31 and Gli1. The representative images were displayed in (**D**) and the statistical data were showed in (**E**). **F** The IF assay to observe the pericyte coverage of blood vessel. The CD31 antibody and α-SMA were used to recognize endothelia cells and preicyte, respectively. **G** The results of dextran leakage assay. *n* = 8. **H-N** Gli1 overexpression promoted tumor growth (**H**-**J**) and tumor angiogenesis (K-L), and increased pericyte coverage of vascular (**M**) and dextran leakage (**N**) in the xenograft of NCI-H460 cells. All the data were showed as mean ± SD, *n* = 6. ^*^p < 0.05; ^***^p < 0.001 vs A549^NC vector^ or NCI-H460^NC vector^ cells

We next confirmed these findings with a xenograft model (Fig. 2A). Compared with the A549^NC vector^ group, the A549^Gli1 vector^ group exhibited increased tumor growth,

as revealed by the tumor growth curve, tumor weight statistics and tumor appearance (Fig. 2B-C and Supporting Fig. 3). Pathological analysis showed that the necrotic area in A549^Gli1 vector^ tumors was reduced when compared with that in A549^NC vector^ tumors, as was the Ki67 index and CD31 staining (Fig. 2D-E). IF assay also revealed that αSMA positive pericyte coverage around the CD31 positive cells were obviously increased in A549^Gli1 vector^ tumors (Fig. 2F). In addition, the mice were injected with 70 kDa rhodamine-labeled dextran to observe tumor vascular permeability. The results showed that vascular permeability was dramatically increased (Fig. 2G). Similar results were obtained with NCI-H460 cells. Tumors in the NCI-H460^Gli1 vector^ cells group tended to be larger (Fig. 2H-J) and have a higher Ki67 index, a greater microvessel density (Fig. 2K-L), greater αSMA-positive pericyte coverage surrounding CD31-positive cells (Fig. 2M), and more dextran leakage (Fig. 2N). These results strongly suggest that Gli1 overexpression promotes tumor angiogenesis and increases pericyte coverage.

**Fig. 3:**
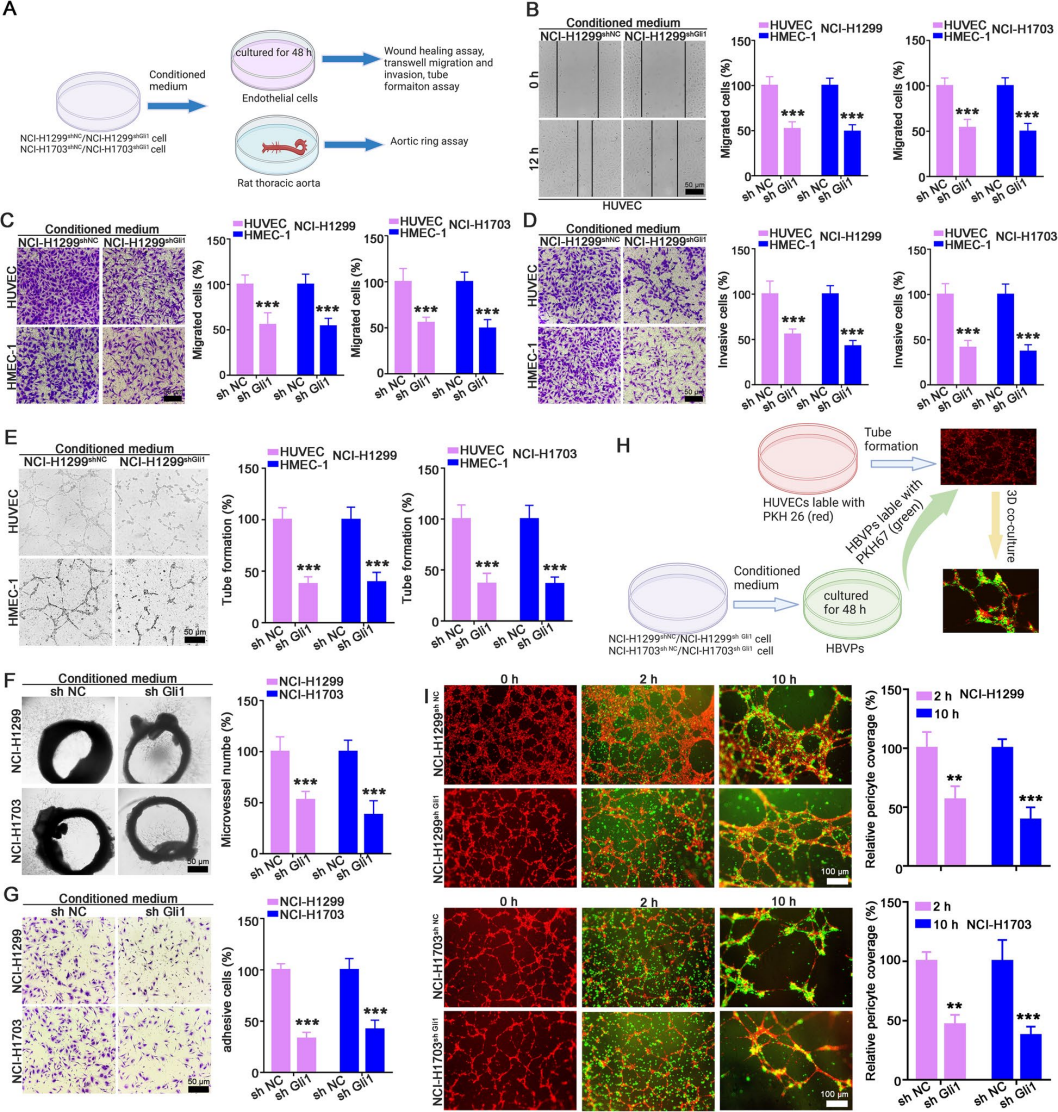
The CM from Gli1-knockdown NSCLC cells suppressed angiogenesis in vitro and ex vivo. **A** Schematic strategy of endothelial cells and aorta rings treated with CM from Gli1-knockdown NSCLC cells. **B-E** The CM from NCI-H1299^shGli1^ cells or NCI-H1703^shGli1^ cells suppressed the migration (**B** and **C**), invasion (**D**) and tube formation (**E**) abilities of HUVECs and HMEC-1 cell. **F** The CM from NCI-H1299^shGli1^ cells or NCI-H1703^shGli1^ cells reduced vessel sprouting in the rat aorta rings. **G-I** The CM from NCI-H1299^shGli1^ cells and NCI-H1703^shGli1^ cells inhibited HBVP adhesion (**G**) and recruitment to newly blood vessel (H and I). All the data were showed as mean ± SD, *n* = 3. ^**^p < 0.01; ^***^p < 0.001 vs NCI-H1299^sh NC^ or NCI-H1703^sh NC^ cells

### Genetic inhibition of Gli1 in NSCLC cells impaired tumor angiogenesis

To further verify the function of Gli1 in angiogenesis, we used the CM of NCI-H1299^shNC^/NCI-H1299^shGli1^ and NCI-H1703^shNC^/NCI-H1703^shGli1^ cells to educate endothelial cells and rat thoracic aortas (Fig. 3A). The results demonstrated that Gli1 silencing in NCI-H1299 and NCI-H1703 cells posed passive effect on tumor angiogenesis-related processes. CM from NCI-H1299^shGli1^ and NCI-H1703^shGli1^ cells obviously reduced the migration (Fig. 3B-C and Supporting Fig. 4 A-B), invasion (Fig. 3D and Supporting Fig. 4C), and tube formation (Fig. 3E and Supporting Fig. 4 D) of HUVECs and HMEC-1 cells. Consistently, the aortic ring assay verified that CM from NCI-H1299^shGli1^ and NCI-H1703^shGli1^ cells also significantly reduced the microvessel density surrounding the aortic ring (Fig. 3F). Moreover, CM from NCI-H1299^shGli1^ and NCI-H1703^shGli1^ cells also obviously decreased the adhesion ability of HBVPs (Fig. 3G), and the HBVPs recruitment to the endothelial capillaries (Fig. 3H-I).

**Fig. 4:**
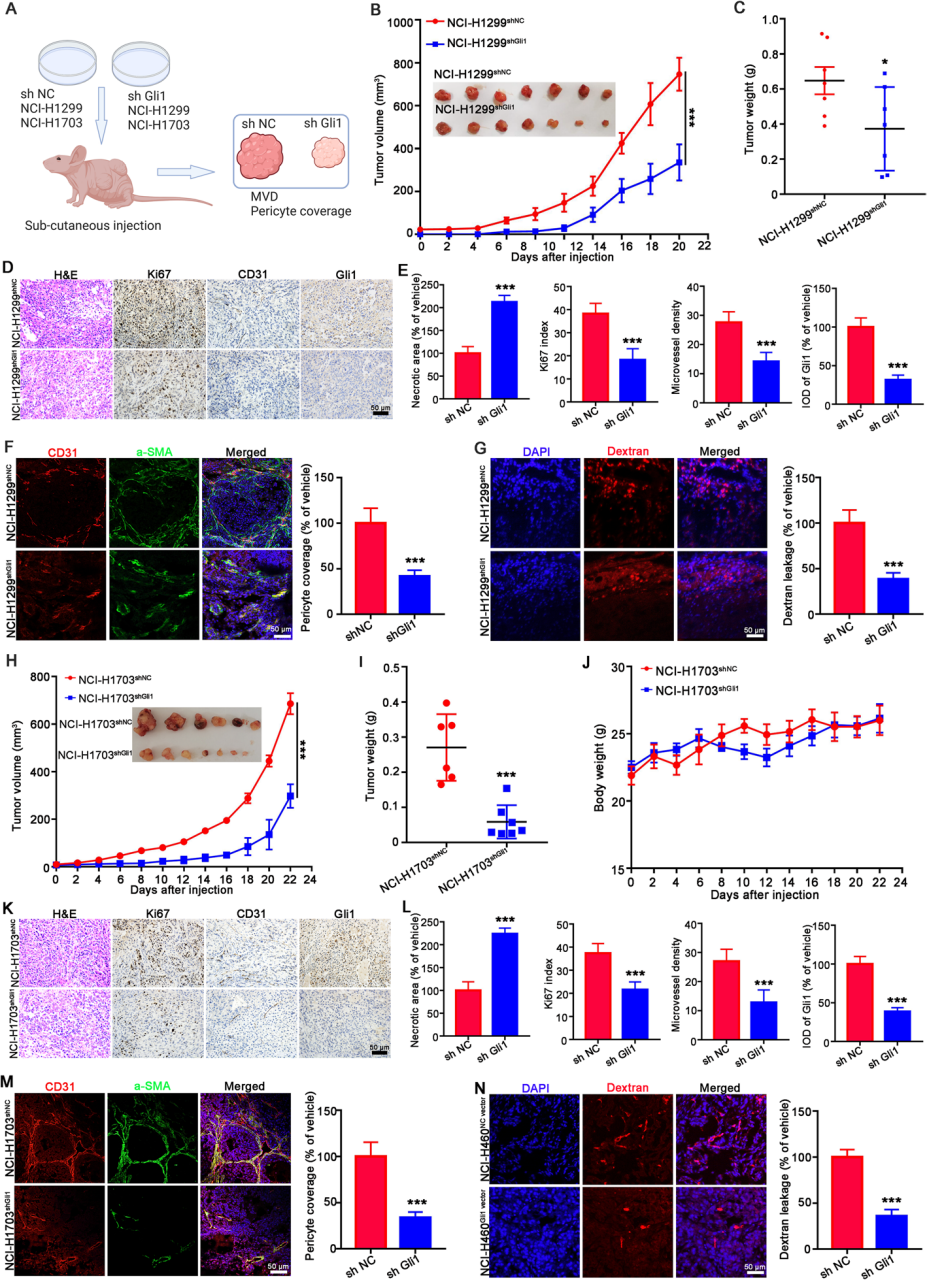
Genetic inhibition of Gli1 attenuated tumor growth and tumor angiogenesis. **A** Flowchart of the protocol to evaluate whether Gli1-knockdown NCI-H1299 or NCI-H1703 cells affect tumor angiogenesis and pericyte coverage. **B**, **C** Gli1-knockdown NCI-H1299 cells attenuated tumor growth. The tumor volume (**B**) and tumor weight statistics (**C**) were showed. **D**, **E** Gli1 silencing in NCI-H1299 cells induced large necrotic area, less Ki67 positive cells and reduced microvessel density in xenograft. The representative images of IHC and quantitative data were showed in (**D**) and (**E**). **F**, **G** Genetic inhibition of Gli1 in NCI-H1299 cells reduced pericyte coverage and dextran leakage in xenograft tissues, *n* = 7. **H-N** Gli1 knockdown in NCI-H1703 cells suppressed tumor growth (H-J), decreased microvessel density (K and L) and reduced pericyte coverage of blood vessel (M), and dextran leakage (N). All the data were showed as mean ± SD, *n* = 6. ^**^p < 0.01; ^***^p < 0.001 vs NCI-H1299^sh NC^ or NCI-H1703^sh NC^ cells

 NCI-H1299^shNC^/NCI-H1299^shGli1^ cells were injected subcutaneously into the nude mice to establish a xenograft model, and the effect of Gli1 on tumor angiogenesis was further evaluated (Fig. 4A). The tumor growth of NCI-H1299^shGli1^ was remarkably suppressed, indicated by the tumor volume curve, tumor appearance and tumor weight statistics (Fig. 4B-C and Supporting Fig. 5). A larger necrotic area and fewer Ki67 + cells and microvessels were detected in the NCI-H1299^shGli1^ xenografts (Fig. 4D-E). IF analyses and permeability assays also revealed less coverage of αSMA-positive pericytes surrounding CD31-positive cells (Fig. 4F) and less dextran leakage (Fig. 4G) in xenografts formed from NCI-H1299^shGli1^ cells. These results were confirmed in NCI-H1703^shNC^/NCI-H1703^shGli1^ xenografts. The growth of tumors generated from NCI-H1703^shGli1^ cells was obviously attenuated (Fig. 4H-J), accompanied by larger necrotic area, a lower Ki67 index and less CD 31 staining blood vessels (Fig. 4K-L). Furthermore, the pericyte coverage and dextran leakage were obviously attenuated in NCI-H1703^shGli1^ tumors (Fig. 4M-N). Taken together, these results indicate that genetic inhibition of Gli1 in NSCLC obviously impairs the angiogenesis-related processes and blood vascular function.

**Fig. 5:**
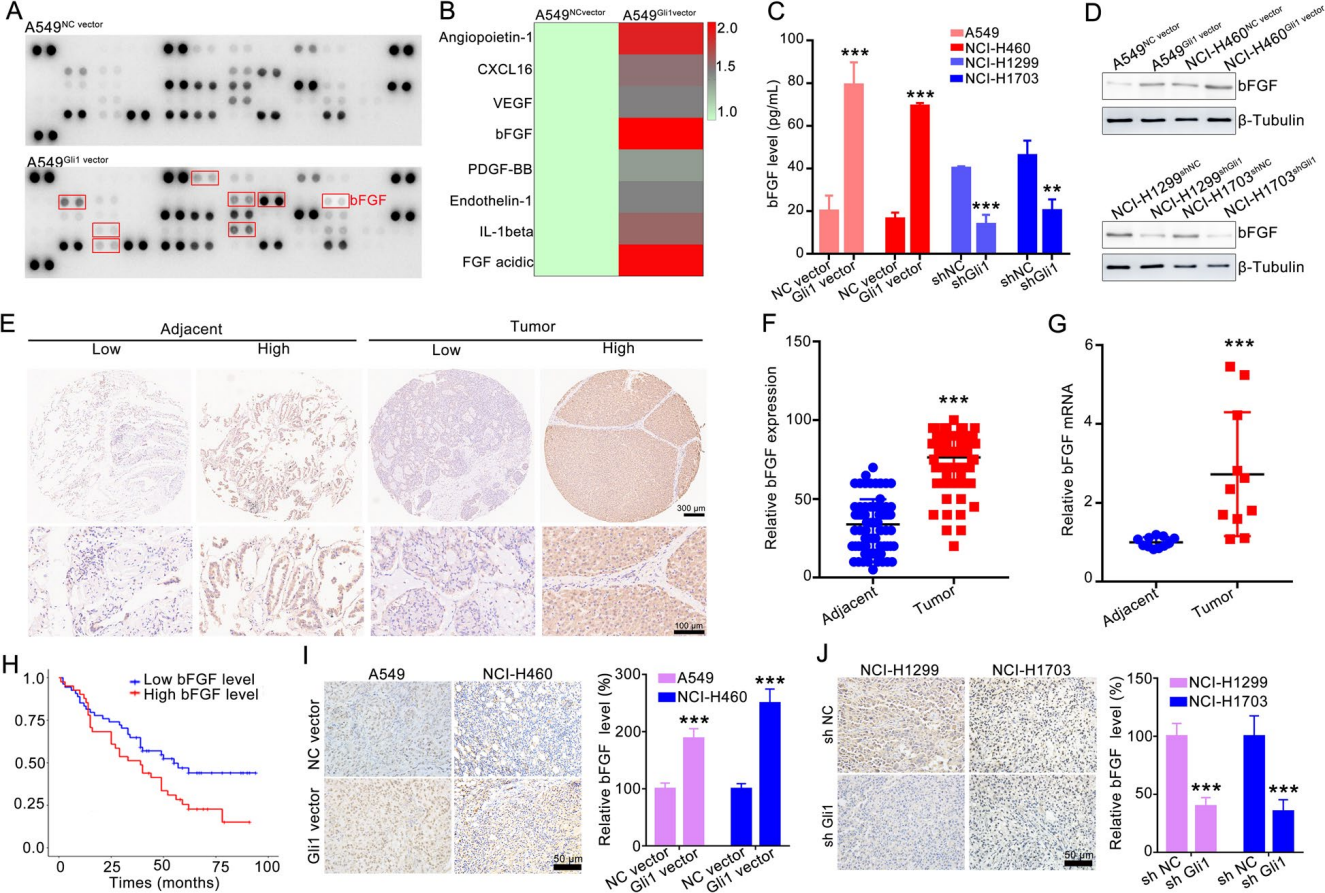
bFGF was involved in Gli1-mediated enhancement effect on tumor angiogenesis. **A** Human angiogenesis array analysis of CM from A549^NC vector^ or A549^Gli1 vector^ cells. And the red box indicates the 8 significant up-regulated protein in CM of A549^Gli1 vector^ cells when compared with that of A549^NC vector^ cells. **B** Summary of Human angiogenesis array analysis of relative signal intensity of upregulated cytokines. **C** The bFGF protein level in the CM of four paired Gli1-overexpressing or Gli1-knockdown cells were detected by ELISA assay, *n* = 3. **D** Western blotting detected the bFGF expression in 4-paired Gli1-overexpressing or Gli1-knockdown NSCLC cells, *n* = 3. **E**–**F** The bFGF expression in 80-paired NSCLC tumor tissues and their corresponding noncancerous lung tissues were detected by IHC. The representative images were showed in (E) and the statistical data were displayed in (F). ^***^p < 0.001 vs adjacent tissues. **G** RT‒qPCR assay determined the bFGF mRNA in 11-paired NSCLC tumor tissues and their corresponding adjacent tissues. ^***^p < 0.001 vs adjacent tissues. **H** Kaplan–Meier analysis of NSCLC patients with a high bFGF expression and a low bFGF expression. **I-J** bFGF expression in xenograft tumors of 2-paired Gli1-overexpressing NSCLC cell lines and 2-paired Gli1-knockdown NSCLC cells detected by IH. All the data were showed as mean ± SD. ^***^p < 0.001 vs NC vector group or shNC group

### Basic fibroblast growth factor (bFGF) is necessary for the Gli1-mediated effects on angiogenesis

To identify Gli1-regulated angiogenic factors in NSCLC cells, a human angiogenic antibody array was applied to determine the differences in angiogenesis-related protein expression between A549^NC vector^ and A549^Gli1 vector^ cells. Interestingly, several proteins, including angiopoietin-1, CXCL16, endothelin-1, bFGF, VEGF, PDGF-BB, IL-1 beta and FGF acidic were obviously upregulated in A549^Gli1 vector^ cells. Among them, bFGF showed the greatest upregulation (Fig. 5A-B). ELISA assay and Western blotting confirmed that Gli1 increased bFGF expression and secretion (CM), whereas Gli1 silencing decreased bFGF expression and secretion (Fig. 5C-D). The results of IHC staining of tissue microarray (containing 90-paired normal lung tissues and NSCLC tissues) showed that bFGF expression in NSCLC tissues was obviously greater than that in normal tissues (Fig. 5E-F and Supporting Fig. 6). RT‒qPCR analysis of 11 representative paired NSCLC tissues and normal tissues also confirmed that bFGF expression was markedly increased in NSCLC tissues (Fig. 5G). Kaplan–Meier analysis revealed that NSCLC patients with a high bFGF level had a shorter overall survival times than those with low a bFGF level (Fig. 5H). In addition, we found that the bFGF level in Gli1-overexpressing xenografts was obviously greater than that in Gli1-negative silenced xenografts (Fig. 5I). Conversely, bFGF expression reduced in xenograft tumors formed from NCI-H1299^shGli1^ and NCI-H1703^shGli1^ cells (Fig. 5J). Taken together, these results indicate that Gli1 may promote tumor angiogenesis by positively regulating bFGF expression.

**Fig. 6:**
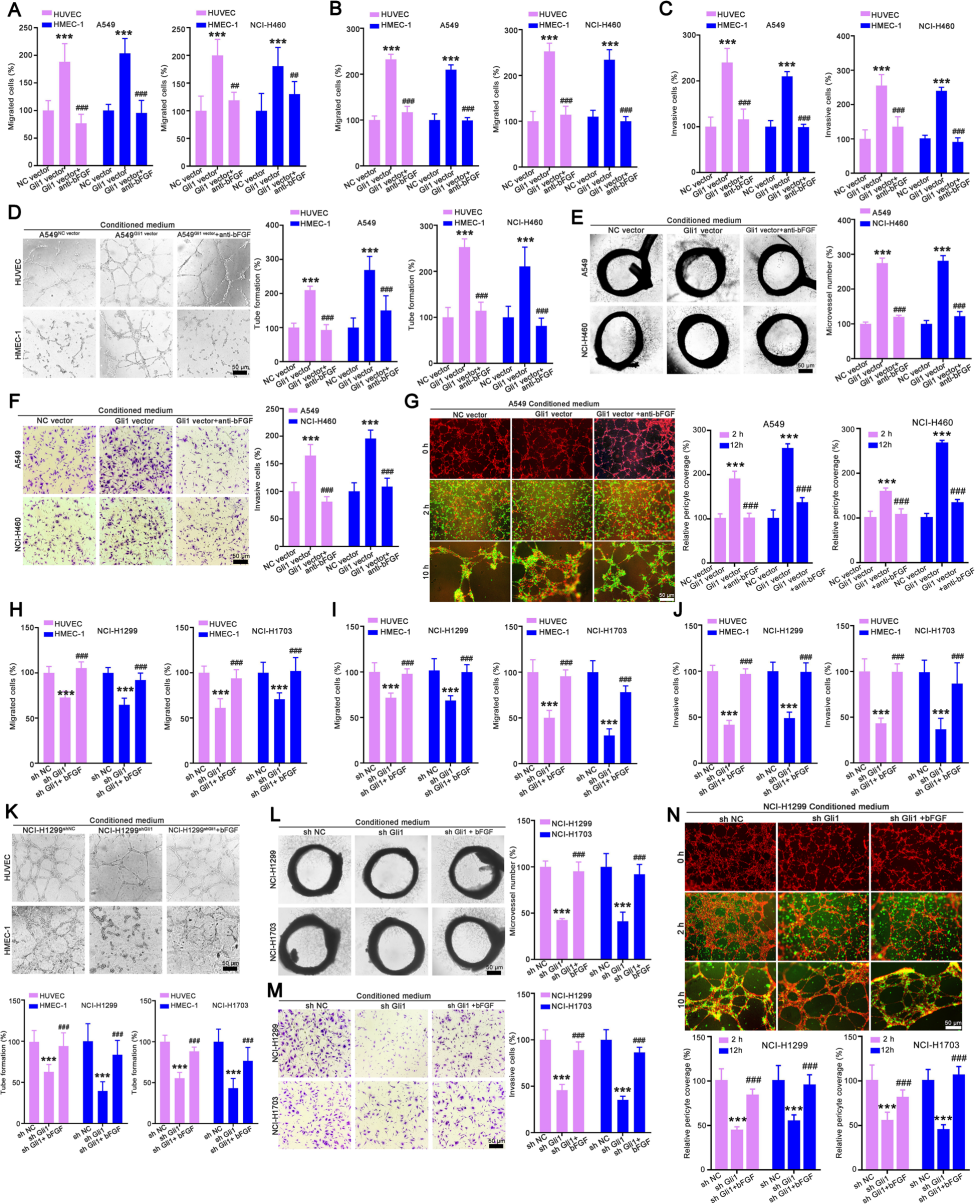
bFGF was critical for Gli1 mediated enhancement effect on angiogenesis and pericyte coverage. **A-D** Anti-bFGF treatment abolished the simulative effect of CM from A549^Gli1 vector^ or NCI-H460^Gli1 vector^ cells on the migration, invasion and tube formation of endothelial cells, indicating by wound healing (**A**), Transwell migration (**B**) and Transwell invasio (**C**), and tube formation assays (**D**). **E** Anti-bFGF attenuated the enhancement effect of CM from A549^Gli1 vector^ or NCI-H460^Gli1 vector^ cells on vessel sprouting. **F**, **G** bFGF neutralizing antibody weaken Gli1-mediated enhancement effect on the adhesion (**F**) and recruitment of HBVP (**G**). **H**–**K** bFGF supplement rescued CM from Gli1-knockdown NSCLC cells mediated inhibitory effect on the migration (**H** and **I**), invasion (**J**) and tube formation (**K**) of endothelial cells. **L** bFGF rescued the vessel sprouting of aortic ring educated by CM of NCI-H1299^sh Gli1^ and NCI-H1703^sh Gli1^ cells. **M**–**N** bFGF treatment enhanced the adhesion (M) and recruitment (N) of HBVP that were treated with CM from NCI-H1299^sh Gli1^ or NCI-H1703^sh Gli1^ cells. All the data were showed as mean ± SD, *n* = 3. ^***^p < 0.001 vs NC vector group or shNC group; ^*###*^*p* < 0.001 vs Gli1 vector group or shGli1 group

To verify the necessity of bFGF as a downstream target of Gli1 to mediate angiogenesis, we used a neutralizing antibody to block secreted bFGF in the CM of A549^Gli1vector^ and NCI-H460^Gli1 vector^ cells. The wound healing, Transwell migration and Transwell invasion assay validated that the Gli1-mediated increases in the migration, invasion and tube formation of HUVECs and HMEC-1 cells were obviously attenuated by anti-bFGF antibody treatment (Fig. 6A-D and Supporting Fig. 7 A-D). Anti-bFGF antibody treatment decreased the extent to which A549^Gli1 vector^ and NCI-H460^Gli1 vector^ cells increased aortic ring sprouting (Fig. 6E). Moreover, similar results were obtained in the pericyte adhesion and 3D-coculture assays. Supplementation with CM from A549^Gli1 vector^ and NCI-H460^Gli1 vector^ cells significantly enhanced the adhesion and recruitment of HBVPs to capillaries, but anti-bFGF antibody treatment obviously attenuated these effects (Fig. 6F-G and Supporting Fig. 7E). We also supplemented the CM of NCI-H1299^shGli1^ and NCI-H1703^shGli1^ cells with recombinant bFGF to further verify the effect of bFGF. The wound healing, Transwell migration and Transwell invasion, and tube formation assays showed that CM from NCI-H1299^shGli1^ and NCI-H1703^shGli1^ cells obviously reduced the migration, invasion and tube formation of endothelial cells. This loss of in vitro angiogenesis was restored when bFGF was added to the CM of NCI-H1299^shGli1^ and NCI-H1703^shGli1^ cells (Fig. 6H-K and Supporting Fig. 8A-D). Consistently, the aortic ring assay (Fig. 6L), HBVP adhesion assay (Fig. 6M) and coculture assay with endothelial (Fig. 6N and Supporting Fig. 8E) also confirmed these results. Collectively, these findings clearly suggest that bFGF is a critical downstream mediator of Gli1 in mediating angiogenesis and blood vessel maturation.

**Fig. 7:**
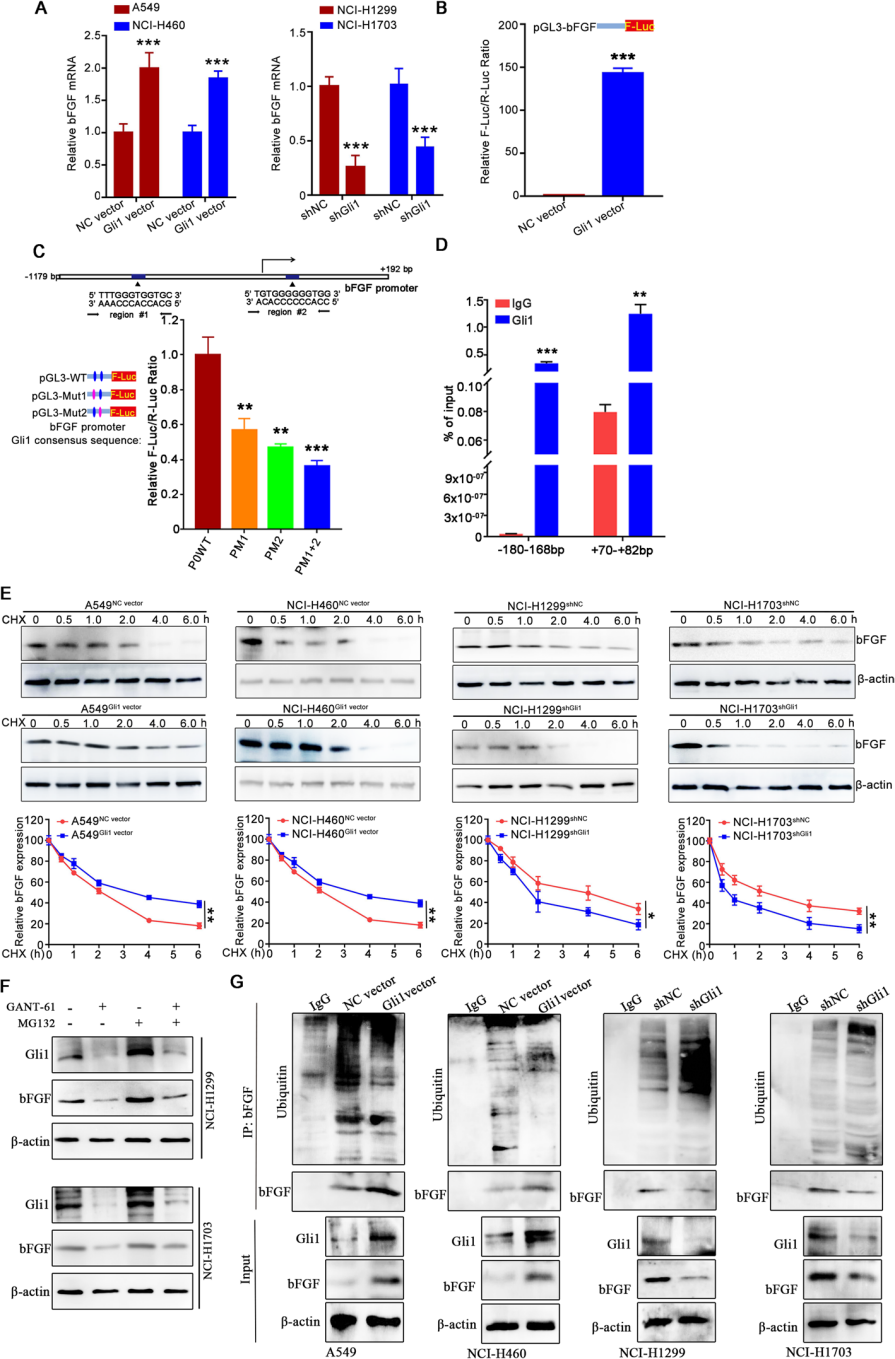
Gli1 regulated bFGF expression by enhancing bFGF transcriptional activity and reducing bFGF protein degradation. **A** Gli1 positively regulated bFGF mRNA level. ^***^p < 0.001 vs NC vector group or sh NC group. **B** Gli1 enhanced the luciferase of bFGF promoter. ^***^p < 0.001 vs NC vector group. **C** Dual-luciferase assay showed that Gli1 regulated bFGF expression by binding to its promoter. ^**^p < 0.01, ^***^p < 0.001vs P0WT group. **D** The result of ChIP-PCR assay. ^**^p < 0.01, ^***^p < 0.001vs IgG group. **E** The effect of Gli1 on bFGF protein stability. Cells were treated with CHX (2 μM) for 0–6 h, and the cells were collected and subjected for Western blotting. ^***^p < 0.001vs NC vector or shNC group. **F** The effect of Gli1 on the degradation of bFGF. **G** The effect of Gli1 on the ubiquitinations of bFGF. All the data were showed as mean ± SD, *n* = 3

**Fig. 8:**
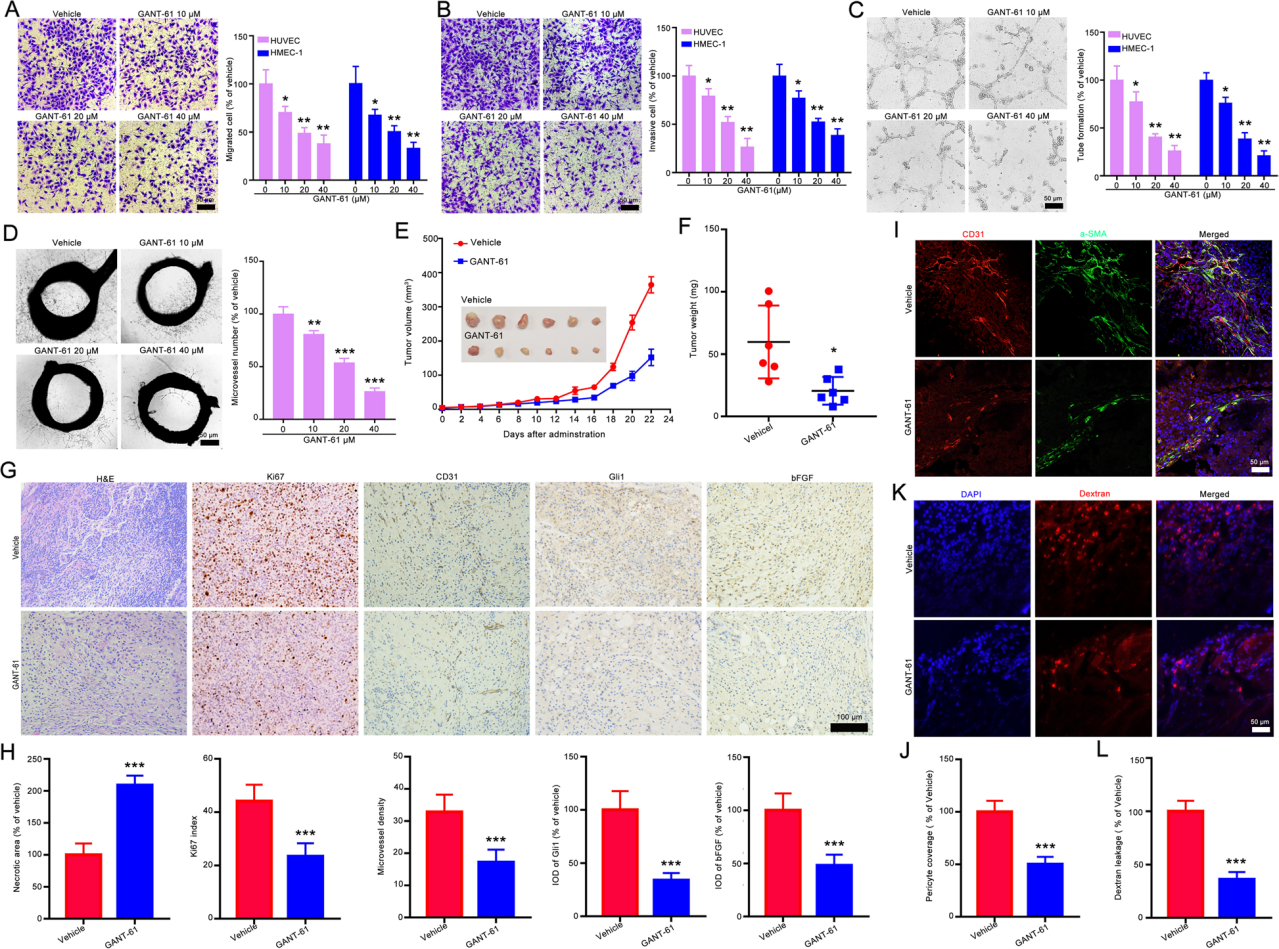
GANT-61 suppressed tumor angiogenesis. **A-C** The effect of GANT-61 on the migration (**A**), invasion (**B**) and tube formation abilities (**C**) of endothelial cells. **D** The effect of GANT-61 on the vessel formation measured by aortic ring assay. *n* = 3. **E**–**H** The effect of GANT-61 on the tumor growth and angiogenesis. When the tumor volume reached approximately 50 mm^3^, the tumor bearing mice were randomly intraperitoneally injected with vehicle or GANT-61 (25 mg/kg) every two day for a total of 24 days. At the end of the experiment, the mice were sacrificed and the tumors were removed, weighted and photographed. The tumor volume curve and the images of tumors were showed in (**E**), the tumor weight statistics was presented in (**F**), the representative images of IHC assay and the quantitative data were displayed in (**G**) and (**H**). **I**,** J** The effect of GANT-61 on the pericyte coverage of blood vessel in NCI-H1299 xenografts. **K**,** L** The effect of GANT-61 on the dextran leakage of tumor vascular. All the data were showed as mean ± SD, *n* = 6. ^*^P < 0.05, ^**^P < 0.01 and ^***^P < 0.001 compared with the vehicle group

### Gli1 increases the bFGF protein by regulating bFGF transcriptional activity and protein stability

To elucidate the link between Gli1 and the transcription of bFGF, several experiments were conducted. RT‒qPCR analysis showed that Gli1 positively regulated bFGF mRNA expression. The bFGF mRNA level was dramatically upregulated in A549^Gli1 vector^ and NCI-H460^Gli1 vector^ cells. Conversely, the bFGF mRNA level was obviously reduced in the counterpart Gli1-knockdown cells (Fig. 7A). The dual luciferase reporter assay showed that Gli1 overexpression significantly enhanced bFGF promoter-driven luciferase activity (Fig. 7B). Two potential Gli1-binding sites in the bFGF promoter region, encompassing bp -180 to -168 and bp + 70 to + 82 (Supporting Table 2), were identified. We mutated these two binding sites and found that luciferase activity in the pGL3-bFGF-promoter^Mut1−Luc^ and pGL3-bFGF-promoter^Mut2−Luc^ groups was strongly decreased in comparison to that in the pGL3-bFGF-promoter^WT−Luc^ group (Fig. 7C). Additionally, the ChIP-PCR assay revealed that the relative enrichment of the bFGF promoter in the anti-Gli1 group was significantly greater than that in the anti-IgG group (Fig. 7D). These results suggest that Gli1 regulates bFGF transcription.

As bFGF is a highly labile protein [22], we also evaluated the effect of Gli1 on bFGF protein stability. Cells were stimulated with a nontoxic concentration of CHX for 0–6 h to block nascent protein synthesis, after which bFGF expression was analyzed via Western blotting. The results showed that Gli1 overexpression strikingly stabilized the bFGF protein levels. The bFGF protein in A549^NC vector^ and NCI-H460^NC vector^ cells was rapidly degraded, decreasing to an undetectable level within 4 h, but the bFGF protein was detectable until the 6 h time point in A549^Gli1 vector^ and NCI-H460^Gli1 vector^ cells. Whereas, Gli1 knockdown accelerated this degradation process in NCI-H1299 and NCI-H1703 cells (Fig. 7E and Supporting Fig. 9). In addition, GANT-61 treatment obviously reduced bFGF expression, but treatment with MG132 (a proteasome inhibitor) attenuated this effect (Fig. 7F). Further ubiquitination assays demonstrated that Gli1 overexpression reduced the degradation of bFGF in A549 cells and NCI-H460 cells, but genetic inhibition of Gli1 accelerated bFGF degradation (Fig. 7G). These results indicated that Gli1 stabilized bFGF by repressing the ubiquitin–proteasome pathway. In addition, considering that bFGF/FGFR1 and bFG/FGFR2 play different roles in endothelial cells and pericytes, we also evaluated the effect of CM on the bFGF/FGFR1 and bFG/FGFR2 signaling pathways. The results showed that the CM from A549^Gli1 vector^ and NCI-H460^Gli1 vector^ cells significantly increased the levels of bFGF and FGFR1 in endothelial cells, and upregulated bFGF and FGFR2 expression in HBVPs. However, CM from NCI-H1299^shGli1^ and NCI-H1703^shGli1^ cells decreased the effect on FGFR1 and FGFR2 levels (Supporting Fig. 10). All these suggest that Gli1 may regulate bFGF protein expression by affecting its transcriptional activity and degradation.

### Pharmacological inhibition of Gli1 suppresses tumor angiogenesis in vitro and in vivo

We further evaluated whether GANT-61 affects tumor angiogenesis-related processes. Transwell migration and invasion assays also revealed that GANT-61 significantly reduced the migration and invasion abilities of HUVECs and HMEC-1 cells in a dose- dependent manner (Fig. 8A-B and Supporting Fig. 11A-B). Consistently, we observed that GANT-61 treatment obviously decreased the number of tube-like structures in the tube formation assay (Fig. 8C and Supporting Fig. 11C) and the microvessel density surrounding the aortic rings (Fig. 8D). GANT-61 also remarkably inhibited the growth of NCI-H1299 tumors, as indicated by the tumor volume and tumor weight (Fig. 8E-F). GANT-61 administration led to a larger necrotic area, fewer Ki67-positive cells and CD31-positive cells, and lower Gli1 and bFGF expression (Fig. 8G-H). Notably, GANT-61 also reduced pericyte coverage and dextran leakage, as indicated by the presence of α-SMA-positive cells surrounding CD31-positive cells and the relative fluorescence intensity in the tumor tissues (Fig. 8I-L). Taken together, these findings indicate that GANT-61 significantly suppresses tumor angiogenesis.

## Discussion

**Fig. 9:**
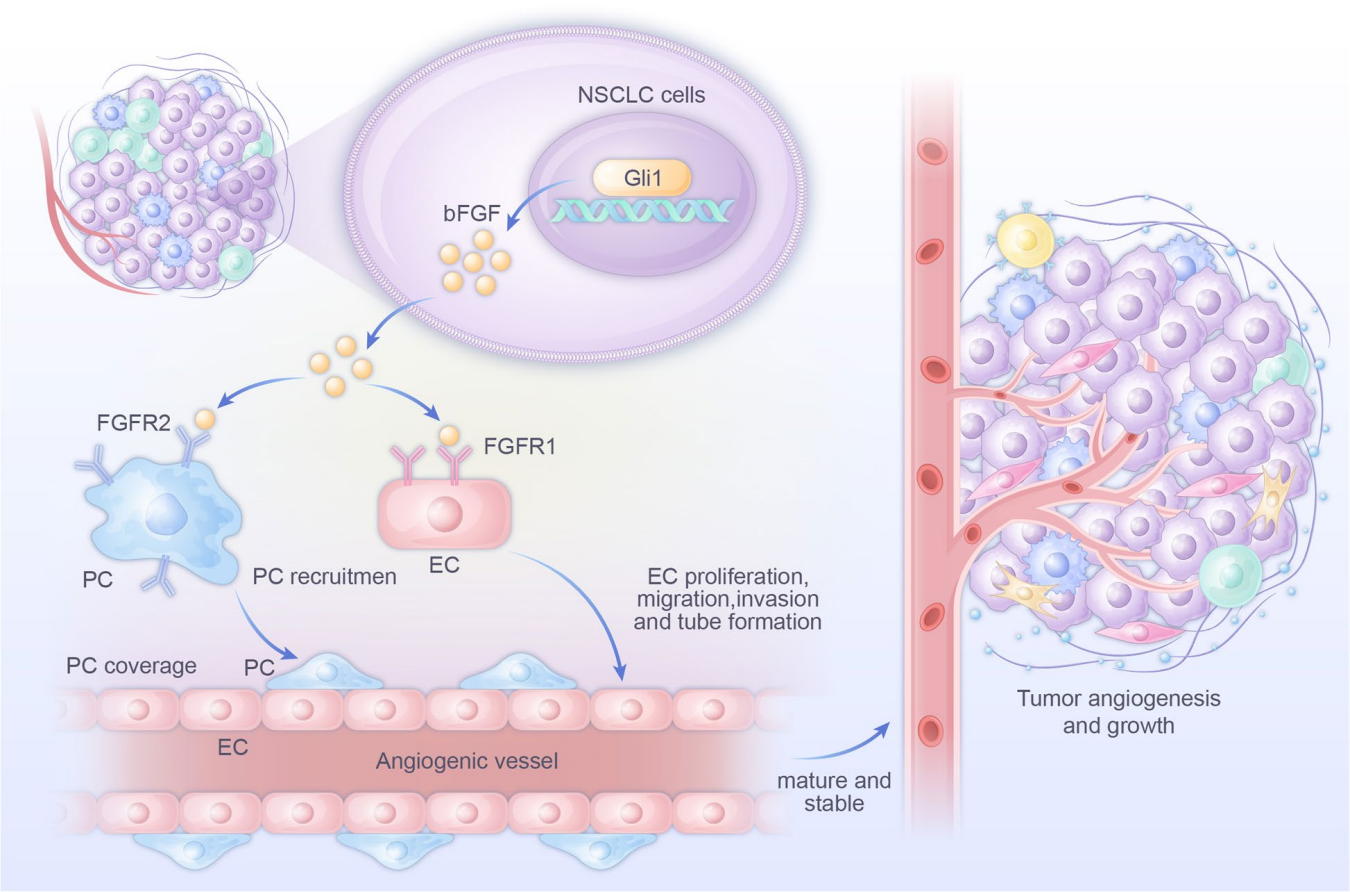
A schema showing that Gli1-mediated tumor cell-derived bFGF promotes tumor angiogenesis by regulating bFGF expression. The bFGF bind with FGFR1 in the endothelial cells and FGFR2 in the pericytes, and then promotes endothelial cell mediated- and pericytes-mediated motility for angiogenesis, leading to tumor angiogenesis and growth of NSCLC

Suppressing tumor angiogenesis is a promising therapeutic strategy for NSCLC, and increasing numbers of AADs have been used for NSCLC therapy. However, the emergence of AAD resistance impedes their further development [23]. Gli1 is a critical regulator of tumorigenesis and invasiveness [24, 25]. Our previous study showed that the Gli1 level was increased in NSCLC tissues and Gli1 enhanced NSCLC metastasis by regulating Snail expression [18]. As angiogenesis is necessary for tumor metastasis, we speculate that Gli1 may be involved in the angiogenesis process in NSCLC. Therefore, we verified that Gli1 overexpression in NSCLC cells promoted angiogenesis. bFGF was identified as a critical regulator of the Gli1-mediated proangiogenic effect. In addition, Gli1 actively regulated the bFGF protein level by promoting bFGF transcriptional activity and reducing bFGF protein degradation (Fig. 9). Moreover, pharmacological inhibition of Gli1 obviously suppressed tumor angiogenesis. In summary, our study provides novel mechanistic insights into the function of Gli1 in regulating NSCLC angiogenesis.

Increasing evidences has confirmed that the crosstalk between NSCLC cells and blood vascular cells is one of most important regulators of AAD resistance. Further elucidation of the underlying molecular mechanisms could aid in the development of therapeutic strategies to overcome AAD resistance [26, 27]. Gli1 is crucial for the activation of Hh/Gli1 signaling pathway. When Gli1 is activated, it translocates to the nucleus, where it leads to the expression of various target genes and activation of downstream signaling pathways [28]. Several studies have suggested that Gli1 contributes angiogenesis. Gli1^+^ cells promote type H vessel formation during the bone defect healing process [29]. Blockade of the Shh/Gli1 signaling pathway significantly suppresses tumor angiogenesis [19] However, the role of Gli1 in mediating the crosstalk between NSCLC cells and blood vascular cells and whether this crosstalk is involved in tumor angiogenesis are still unclear. Our study identified Gli1 as a critical regulator of the crosstalk between NSCLC cells and vascular cells. Gli1 in NSCLC cells promoted the endothelial cell and pericyte motility required for angiogenesis by promoting bFGF expression. In addition, suppressing the Gli1-bFGF axis obviously reduced tumor angiogenesis and growth. Taken together, these findings provide evidence that crosstalk between NSCLC cells and vascular cells is crucial for tumor angiogenesis. Our study also suggests that targeting the crosstalk between lung cancer cells and blood vascular cells might be a potential therapeutic strategy to overcome AAD resistance in NSCLC.

bFGF is one of most important angiogenic factors in the heparin-binding FGF family of growth factors [30, 31]. It exerts its proangiogenic effect by activating FGF receptors (FGFRs), including FGFR1, FGFR2, FGFR3 and FGFR4. bFGF has been demonstrated to play dual roles in angiogenesis. bFGF-FGFR1 signaling promotes endothelial cell proliferation and sprouting. Moreover, bFGF-FGFR2 regulates perivascular contents and increases pericyte coverage, leading to the maturation of blood vessels [32, 33]. In addition, the bFGF-FGFR signaling pathway contributes to resistance to anti-VEGF therapy. Intercellular FGF2 signaling between endothelial cells and pericytes has been demonstrated to be necessary for resistance to anti-VEGF therapy. Moreover, anti-FGF therapy is a potential strategy to compensate for resistance to anti-VEGF therapy [34, 35]. In our study, we reported that bFGF played a prominent role in the communication between NSCLC cells and vascular cells. bFGF is critical for the Gli1-mediated enhancement effect of endothelial cell and pericyte motility to support angiogenesis and blood vessel maturation. Anti-bFGF antibody treatment obviously abolished the Gli1-mediated enhancement of this motility. Whereas, bFGF rescue experiments showed that bFGF attenuated the Gli1 silencing-mediated inhibition of these processes. Gli1 upregulated bFGF expression by promoting its transcriptional activity and suppressing its protein degradation. Take together, the findings of our study provide strong evidence for the role of the Gli1-bFGF axis in regulating NSCLC angiogenesis. Our study also suggests that the intercellular communication axis between NSCLC cells and vascular cells is critical for tumor angiogenesis. In addition, considering the role of bFGF in resistance to anti-VEGF therapy, we speculate that targeting the Gli1-bFGF signaling pathway might be a potential therapeutic strategy for overcoming resistance to antiangiogenic and anti-VEGF therapies in NSCLC. We will further explore this possibility in future studies.

GANT-61 is an inhibitor of Gli1 and Gli2. It inhibits the proliferation of many types of cancer cells proliferation, including glioblastoma, breast cancer and pancreatic cancer cells [36–39]. GANT-61 also inhibits A549 cell proliferation [40]. Our recent study also showed that GANT-61 administration suppressed NSCLC metastasis [18]. However, whether GANT-61 suppresses angiogenesis in NSCLC is still vague. Herein, we showed that GANT-61 obviously suppressed the migration, invasion and tube formation of endothelial cells. Further research proved that GANT-61 treatment obviously suppressed tumor growth and reduced the tumor vascular density and pericyte coverage of blood vessels in NCI-H1299 cell xenografts. GANT-61 treatment also reduced dextran leakage in tumor tissues. These results indicate that GANT-61 is a potential antiangiogenic agent for treating NSCLC.

Altogether, our study demonstrates that the Gli1-bFGF axis is crucial for the crosstalk between NSCLC cells and vascular cells. Our study suggest that Gli1 may be a promising therapeutic target for suppressing angiogenesis in NSCLC. Our findings also provide new mechanistic insights into that the crosstalk between NSCLC cells and vascular cells.

### Supplementary Information

**Supplementary Material 1.**

## Data Availability

The data that support the findings of this study are available from the corresponding author upon reasonable request.

## Abbreviations

NSCLC: Non-small lung cancer
Gli1: Glioma-associated oncogene 1
EGFR: Epidermal growth factor receptor
AAD: Antiangiogenic drug
HUVECs: Human umbilical vein endothelial cell
HMEC-1: Human microvascular endothelial cell-1
HBVPs: Human brain vascular pericytes
CHX: Cycloheximide
ChIP: Chromatin Immunoprecipitation
bFGF: Basic fibroblast growth factor
FGFRs: FGF receptors
