# Supplementary material for: Gli1-mediated tumor cell-derived bFGF promotes tumor angiogenesis and pericyte coverage in non-small cell lung cancer
Source: J Exp Clin Cancer Res. 2024 Mar 16;43:83. doi: 10.1186/s13046-024-03003-0 (PMC10944600; doi:10.1186/s13046-024-03003-0)
Supplement: Supplementary file 1 — Supplementary Material 1. [file 13046_2024_3003_MOESM1_ESM.docx]

Supporting Information for

**Gli1-mediated tumor cell-derived bFGF promotes tumor angiogenesis and pericyte coverage in non-small cell lung cancer**

Xueping Lei^1†^, Zhan Li^1†^, Manting Huang^2†^, Lijuan Huang^1†^, Yong Huang^1^ , Sha Lv^1^, Weisong Zhang^1^, Zhuowen Chen^1^, Yuanyu Ke^1^, Songpei Li^1^, Qianfei Chen^1^, Xiangyu Yang^1^，Qiudi Deng^3*^, Junshan Liu^4*^, Xiyong Yu^1*^

^1^ Guangzhou Municipal and Guangdong Provincial Key Laboratory of Molecular Target & Clinical Pharmacology, the NMPA and State Key Laboratory of Respiratory Disease, College of Pharmacy & The Fifth Affiliated Hospital, Guangzhou Medical University, Guangzhou, 511436, PR China

^2^ Zhongshan Hospital of Traditional Chinese Medicine Affiliated to Guangzhou University of Traditional Chinese Medicine, Zhongshan, 528400, PR, China

^3^ GMU-GIBH Joint School of Life Sciences, The Guangdong-Hong Kong-Macau Joint Laboratory for Cell Fate Regulation and Diseases, Guangzhou Medical University, Guangzhou, 511436, PR, China

^4^ School of Traditional Chinese Medicine, Southern Medical University, Guangzhou 510515, PR China; Guangdong Provincial Key Laboratory of Chinese Medicine Pharmaceutics, Guangzhou 510515. P. R. China.

^*^Corresponding authors.

Tel: +86-20-37103261, Email: yuxycn@gzhmu.edu.cn (XY. Yu).

or Tel: +86-20-61648539; Email: liujunshan@smu.edu.cn (JS. Liu)

or Tel: +86-20-31100902, Email: dengqiudi@gzhmu.edu.cn (QD. Deng).

^†^These authors contribute equally to the work: Xueping Lei, Zhan Li, Manting Huang and Lijuan Huang.

**Supplementary tables**

| **Supplementary table1. RT-PCR primers used in this study.** | | |
| --- | --- | --- |
| Gene | Forward Primer(5'-3') | Reverse Primer(5'-3') |
| bFGF | TATTTCTTTGGCTGCTACTTG | TCCAGCATTTCGGTGTTG |
| bFGF (-180 to -168 bp） | CTATGGGAGGCTGAGGCGG | TTCTCCATGTTGGTCAGGCTG |
| bFGF (+70 to +82 bp） | TTGAGTCACGGCTGGTTGC | TTATCCCCCAAAAGTCACCC |
| GAPDH | CGCTCTCTGCTCCTCCTGTT | CCATGGTGTCTGAGCGATGT |

**Supporting table 2. The information of which Gli1 binds at the promoter of bFG analyze by Analysis of the JASPAR database.**

| TF | Pattern name | Source | Sequence name | Start | Stop | Strand | Score | P value | Q value | Matched motif |
| --- | --- | --- | --- | --- | --- | --- | --- | --- | --- | --- |
| GLI1 |  | database | NC_000004.12:122825690-122826872 | 817 | 828 | + | 15.7551 | 2.31E-06 | 0.00503 | TTTGGGTGGTGC |
| GLI1 |  | database | NC_000004.12:122825690-122826872 | 818 | 827 | - | 14.4771 | 7.84E-06 | 0.0173 | CACCACCCAA |
| GLI1 |  | database | NC_000004.12:122825690-122826872 | 818 | 828 | + | 14.2111 | 9.66E-06 | 0.0216 | TTGGGTGGTGC |
| GLI1 |  | database | NC_000004.12:122825690-122826872 | 1095 | 1106 | + | 11.9694 | 3.58E-05 | 0.0391 | TGTGGGGGGTGG |
| GLI1 |  | database | NC_000004.12:122825690-122826872 | 1096 | 1106 | + | 11.1667 | 5.04E-05 | 0.0564 | GTGGGGGGTGG |
| GLI1 |  | database | NC_000004.12:122825690-122826872 | 1096 | 1105 | - | 11.4954 | 6.04E-05 | 0.0667 | CACCCCCCAC |

**Supporting Figures**

**
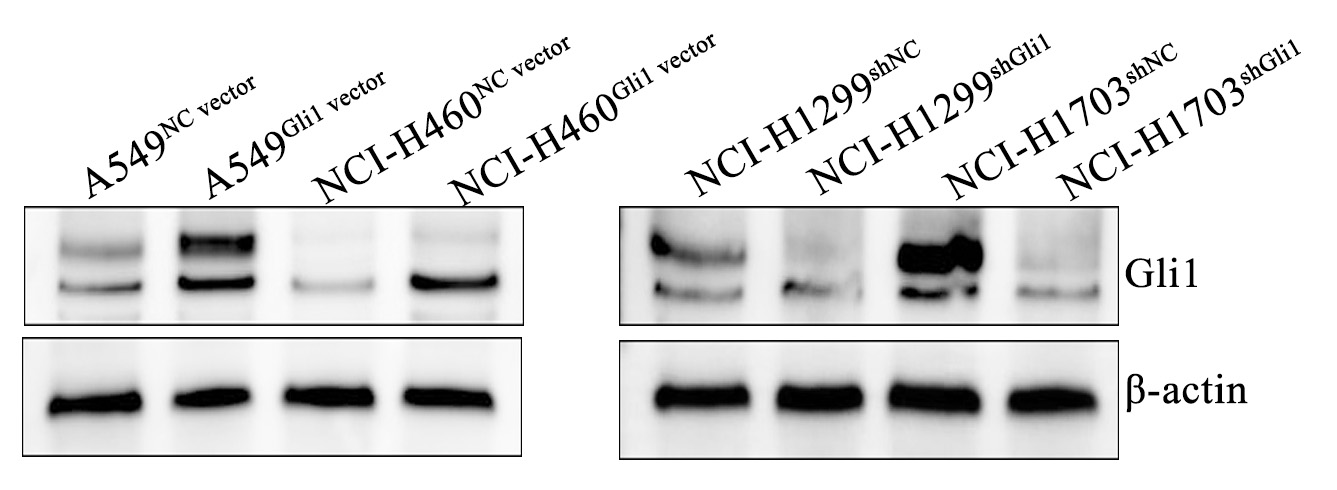
**

**Supporting Fig. 1 The Gli1 expression in A549^NC vector^/A549^Gli1vector^, NCI-H460^NC vector^/NCI-H460^Gli1vector^ cells, NCI-H1299^shNC^/NCI-H1299^shGli1^ and NCI-H1703^shNC^/NCI-H1703^shGli1^ cells detected by Western blotting.**

**
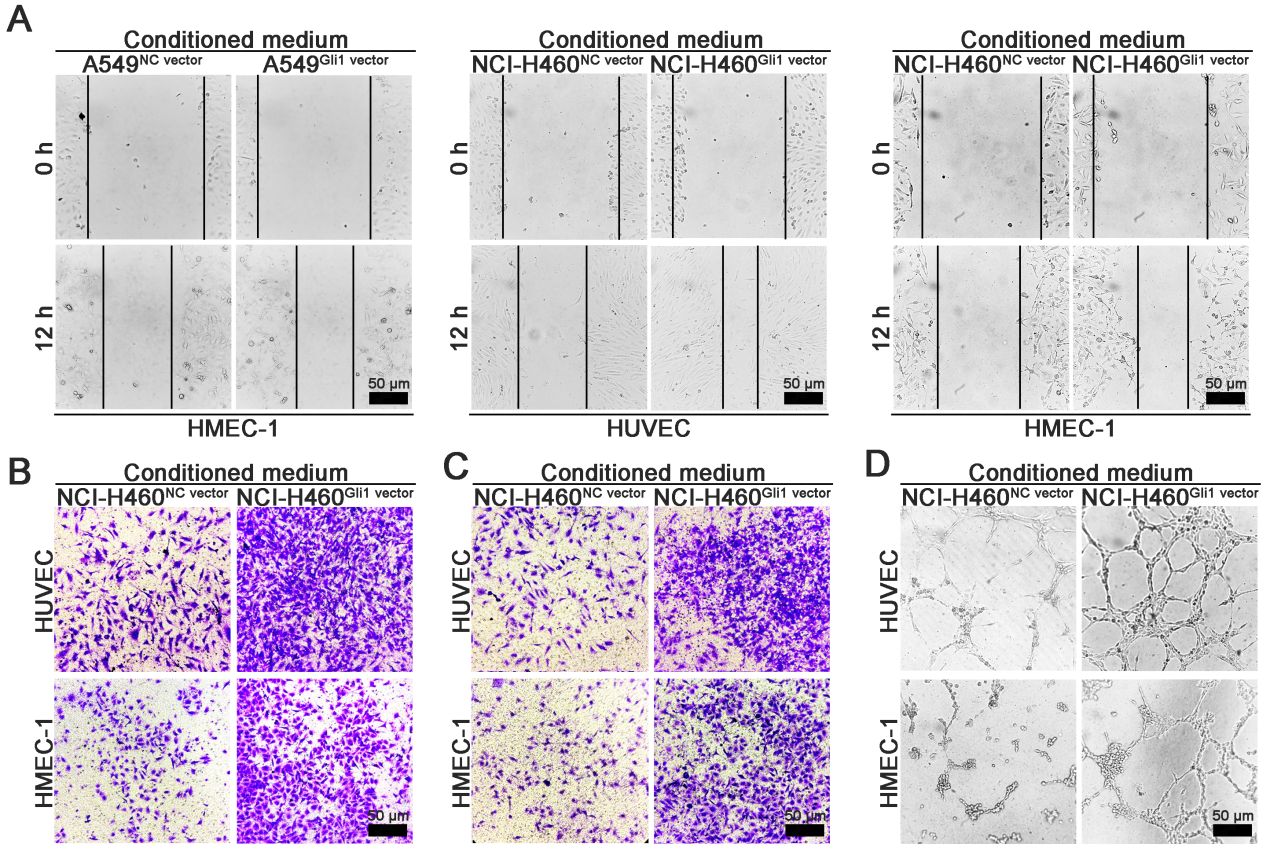
**

**Supporting Fig. 2** The CM of Gli1-overexpressing NSCLC cells enhanced the migration and invasion of A549 and NCI-H460 cells. **A** The representative images of A549 and NCI-H460 cell in wound healing assay. **B-D** The representative images of NCI-H460 cells in Transwell migration (B), Transwell invasion(C) and tube formation (D) assays.


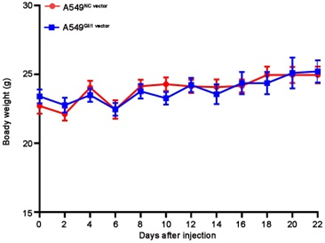


**Supporting Fig. 3 The body weight curve of the mice bearing A549^NC vector^ and A549^Gli1 vector^ tumors.**


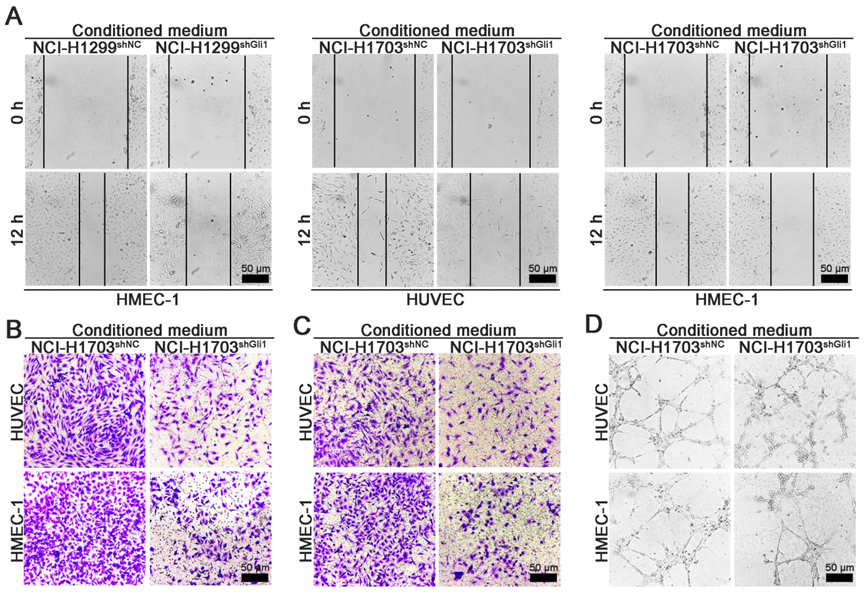


**Supporting Fig. 4** The CM of Gli1-sliencing NSCLC cells attenuated the migration and invasion of NCI-H1299 and NCI-H7703 cells. **A** The representative images of NCI-H1299 and NCI-H703 cell in wound healing assay. **B-D** The representative images of NCI-H1703 cells in Transwell migration (B), Transwell invasion (C) and tube formation (D) assays.


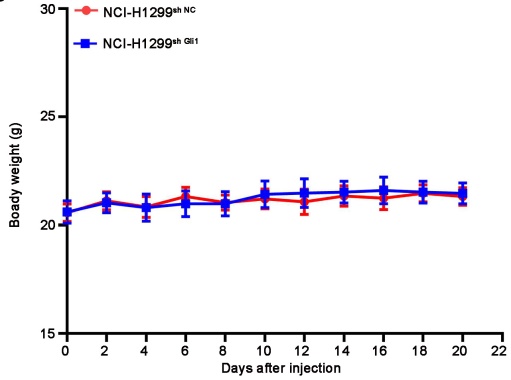


**Supporting Fig. 5 The body weight curve of mice bearing NCI-H1299^shNC^ and NCI-H1299^shGli1^ tumors.**

**
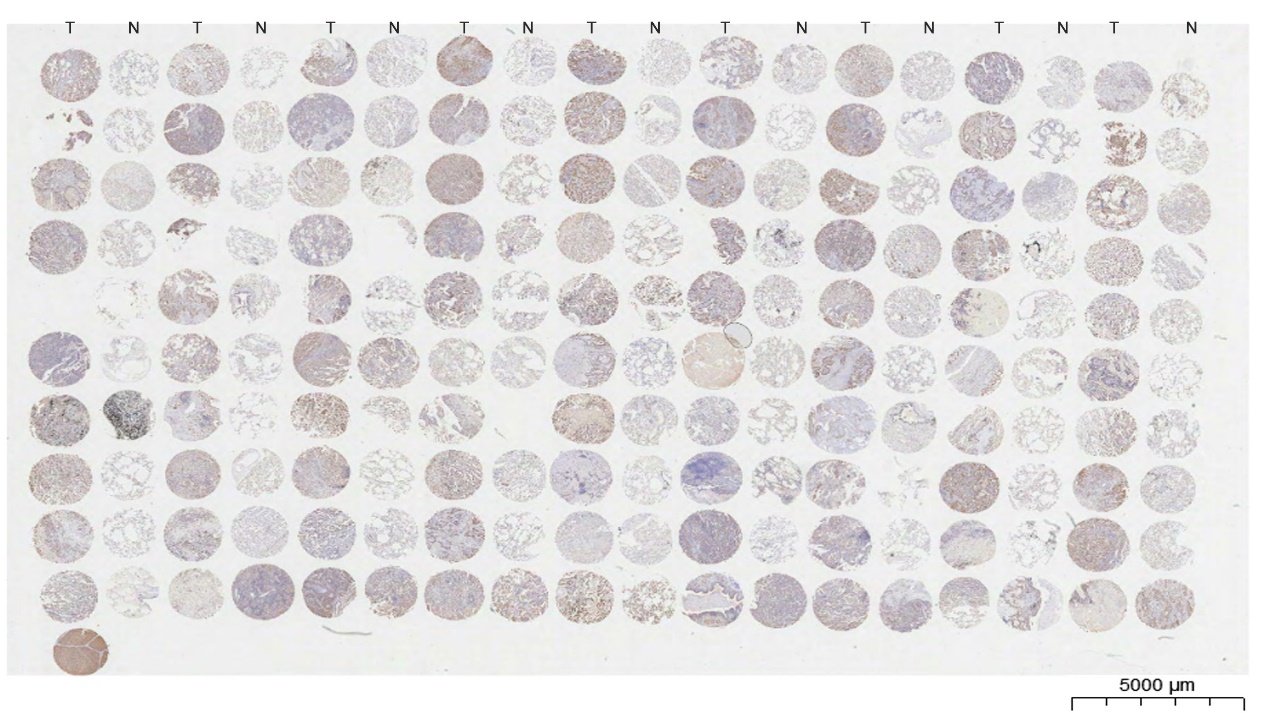
**

**Supporting Fig. 6 The images of the tissue’s microarrays of 80-paired NSCLC tumor tissues and their corresponding noncancerous lung tissues.** T；tumor tissues, N: normal tissues.


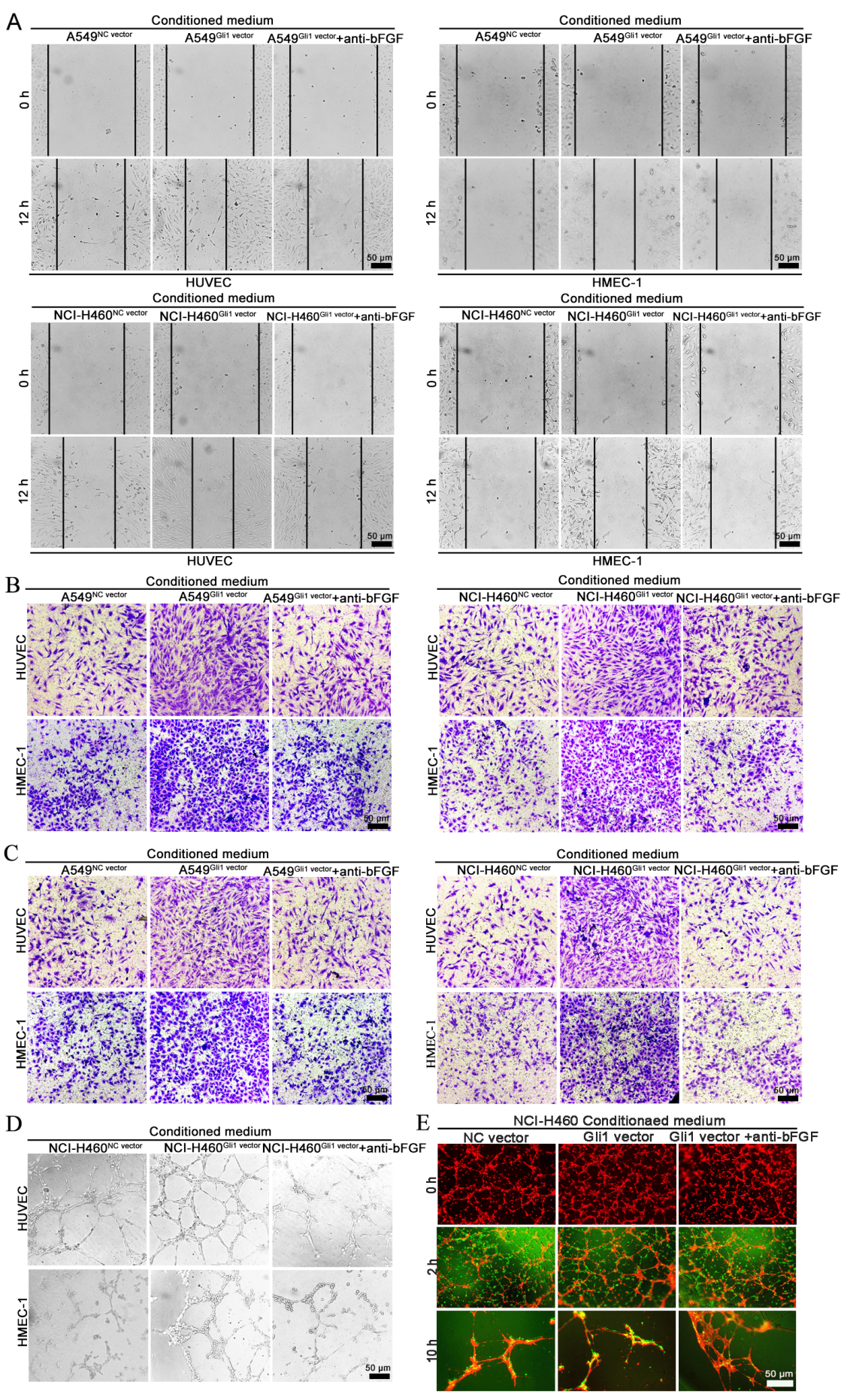


**Supporting Fig. 7 Anti-bFGF treatment attenuated Gli1-meidated enhancement effect on the migration and invasion of A549^Gli1 vector^ and NCI-H460^Gli1 vector^ cells. A** Anti-bFGF treatment alleviated Gli1-mediated enhancement effect on the migration of A549 and NCI-H460 cells detected by wound healing assay. **B-C** Anti-bFGF treatment attenuated the Gli1-mediated accelerative effect on the migration and invasion of A549 and NCI-H460 cells indicated by the transwell migration and invasion assay. **D** Anti-bFGF treatment alleviated the enhancement effect of Gli1 on the bube formation of NCI-H460 cells. **E** Anti-bFGF attenuated Gli1-mediated stimulative effect on pericyte coverage.


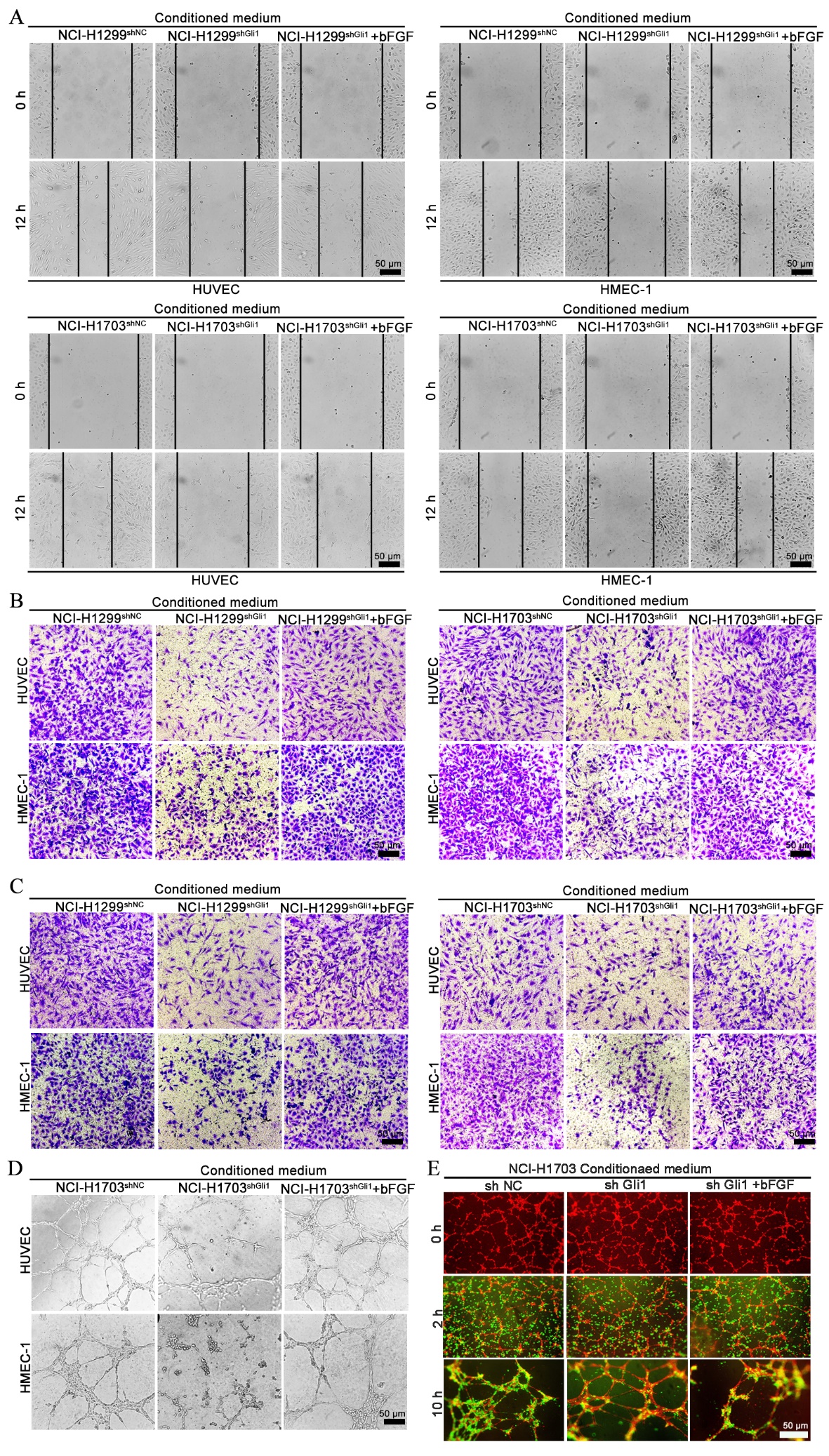


**Supporting Fig. 8 bFGF supplement rescued Gli-silencing mediated inhibitory effect endothelial cells and pericytes educated by NCI-H1299^shGli1^ or NCI-H1703^shGli1^ cells.** The representative images of wound healing (A), Transwell migration (B), Transwell invasion (C)，tube formation (D) and 3D co-culture (E) assays were shown.

**
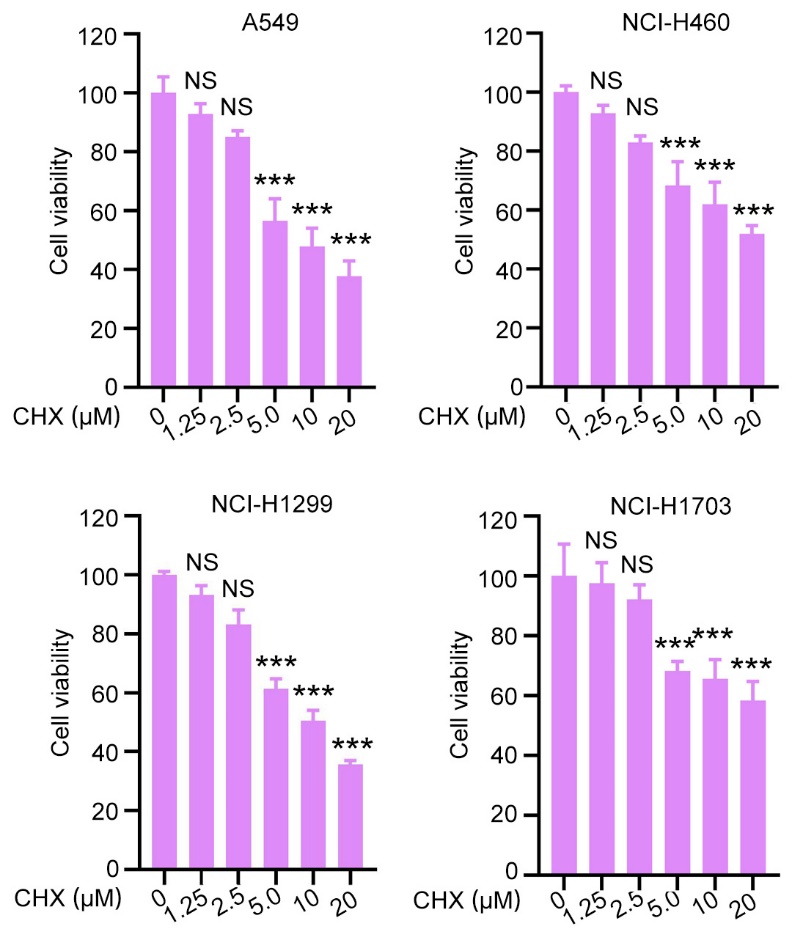
**

**Supporting Fig. 9 The effect of CHX on the cell proliferation of A549, NCI-H460, NCI-H1299 and NCI-H1703 cells.** The cells were treated with various concentration of CHX for 48 h, and the cell viability was measured with CCK-8 assay. The data were showed as mean±SD, n=3. ***p<0.01*; ****p<0.001*, NS：no significant difference.


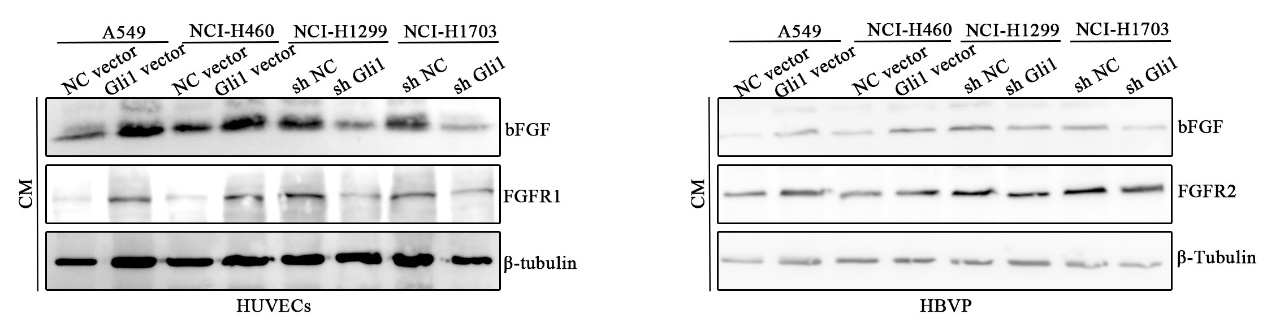


**Supporting Fig. 10 The effect of Gli1 on bFGF/FGFR1 and bFG/FGFR2 signaling pathways.**

**
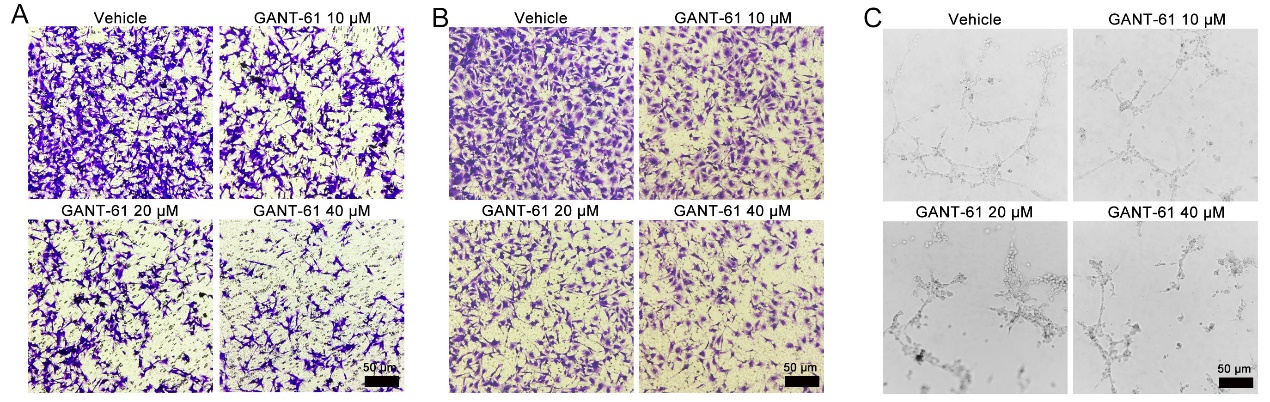
**

**Supporting Fig. 11 The effect of GANT-61 on the migration (A), invasion (B) and tube formation (C) abilities of HMEC-1 cells.** The representative images were shown.
